# Root and mycorrhizal contributions to soil organic carbon changes following 12 years of poplar coppice on former cropland and grassland

**DOI:** 10.1007/s11104-025-07995-2

**Published:** 2025-10-29

**Authors:** Gonzalo Berhongaray, Ivan A. Janssens, M. Francesca Cotrufo, Tim De Meulder, Marilyn Roland, Reinhart Ceulemans

**Affiliations:** 1 ICiAgro Litoral (UNL-CONICET), Facultad de Ciencias Agrarias, Universidad Nacional del Litoral, Kreder 2805 - Esperanza (CP 3080), Esperanza, Argentina; 2 Plant and Ecosystems (PLECO), Department of Biology, University of Antwerp, Antwerp, Belgium; 3 Department of Soil and Crop Sciences, College of Agricultural Sciences, Colorado State University, Fort Collins, CO USA

**Keywords:** Soil carbon, Mycorrhizae, Roots, Carbon accrual, Short-rotation coppice, POPFULL

## Abstract

**Background and Aims:**

The establishment of bioenergy plantations as short-rotation coppice poplar systems has been proposed as a sustainable strategy to mitigate climate change through carbon capture. This study evaluates changes in soil organic carbon (SOC) after 12 years of poplar cultivation on former cropland and grassland in Belgium using repeated soil sampling to assess SOC stock changes and in-growth cores to identify carbon input pathways.

**Methods:**

Using isotope tracing and in-growth cores with treatments excluding roots, mycorrhizae and above-ground inputs, we quantified the contributions of roots, mycorrhizae, and dissolved organic matter to new SOC formation and their interaction with the mineralization of native SOC.

**Results:**

Results showed a significant increase in SOC in former croplands while grasslands experienced a slight SOC reduction, highlighting the influence of previous land use on SOC accrual potential. Root-derived inputs surpassed mycorrhizal contributions to SOC formation although both played a role in achieving a positive SOC balance.

**Conclusion:**

This study underscores the critical role of roots in SOC accumulation and the importance of initial soil conditions when designing SOC accrual strategies through bioenergy plantations.

**Supplementary Information:**

The online version contains supplementary material available at 10.1007/s11104-025-07995-2.

## Introduction

Bioenergy crops, such as poplar based plantations, can support the global energy transition and have therefore been proposed as a promising sustainable land use (Jaafari 2023; Pleguezuelo et al. 2015). Bioenergy has a much smaller carbon footprint than fossil fuel based energy because the CO_2_ emitted during combustion was first removed from the atmosphere and sequestered in growing biomass (Sahoo et al. 2022). Moreover, when combined with carbon capture and storage, bioenergy crops constitute a carbon removal technology that is counted upon to realize negative carbon emissions later this century (Palmer And Carton 2021; Reid et al. 2020; Salas et al. 2024). Apart from the biomass carbon that would be harvested and combusted, bioenergy crops may also store carbon in soils, especially when planted on former croplands depleted in soil carbon (Bell et al. 2020; Osei et al. 2024).

Soil organic carbon (SOC) is a crucial determinant of many soil based ecosystem services as nutrient retention and exchange capacity (Solly et al. 2020), or water infiltration and retention (Fu et al. 2021; Zhao et al. 2023). Nonetheless, agricultural management has led to global declines in SOC stocks that contributed to the widespread degradation of agricultural soils (Peralta et al. 2022; Poeplau And Dechow 2023). Increasing SOC content thus makes sense from a soil quality and protection perspective but by reducing atmospheric carbon dioxide levels, SOC accrual also serves as a climate change mitigation technique (Lal et al. 2021; Sykes et al. 2020).

In managed systems with high rates of biomass extraction SOC content typically depends on soil carbon inputs (Chenu et al. 2019; Xu et al. 2021). However, with the exception of above-ground litter fall, the accurate quantification of poplar-derived carbon inputs to soil is difficult. Soil carbon inputs via fine root turnover (Freschet et al. 2021), exudation, sloughing (Irving et al. 2021), or transfers to mycorrhizal symbionts (Janowski And Leski 2023; Verlinden et al. 2018) are notoriously difficult to quantify. As a result, understanding the contributions of below-ground plant inputs to SOC accrual remains a significant challenge.

SOC dynamics following poplar plantation on former croplands often show an initial loss of SOC due to soil disturbance during land-use change, followed by a gradual increase as the system stabilizes and carbon inputs from the afforested vegetation accumulate over time (Arevalo et al. 2011). The increase in SOC following the establishment of a poplar plantation is predominantly observed in the topsoil layer (0–25 cm) where carbon inputs from litter and root turnover are higher (Georgiadis et al. 2017). On the other hand, afforestation of a pasture over 130 years showed no significant change in SOC stocks, emphasizing the influence of initial soil conditions and land-use history on SOC dynamics (Speckert et al. 2023). Poplar bioenergy crops under the short-rotation coppice (SRC) regime are typically established on former or marginal cropland. Chronosequences and paired-site studies across Europe consistently report SOC increases after cropland-to-SRC conversion (especially in the top 20–30 cm), although the size of the response is site specific and lower on SOC rich soils (Georgiadis et al. 2017). In Germany early increments in SOC stocks were observed under SRC; the regional syntheses likewise report SOC accumulation under poplar SRC on former cropland (Tariq et al. 2018). Multi-site analyses and recent reviews show SRC poplar can raise SOC on suitable croplands and is being re-considered within EU “carbon farming” and renewable-energy strategies (Antoniella et al. 2024; Petersson et al. 2025). In Canada and the U.S. hybrid poplar on former cropland increased SOC over time, with rotation length being critical; the study also showed initial losses followed by recovery, underscoring the need for decadal study periods (Arevalo et al. 2011; Osei et al. 2024; Zheng et al. 2017).

Globally, the area of poplar SRC remains small relative to other land uses, and historical expansion has been slow where policy and markets were weak (e.g., Sweden’s experience; Dimitriou et al. 2011). Interest is renewing in Europe and in parts of North America under net-zero and carbon-farming agendas, with recent assessments, datasets and reviews highlighting SRC’s role for negative-emissions energy and SOC co-benefits—especially on marginal or low-SOC cropland. A key mechanistic reason why SRC performs best on low-SOC cropland is the carbon-saturation concept: soils closer to their stabilization capacity respond weakly to extra inputs, whereas C-poor cropland has room to store more C as mineral-associated organic matter (Stewart et al. 2009). This helps explain why conversions from cropland often accrue SOC faster than conversions from already C-rich grassland.

Here, we set out to study changes in poplar-derived carbon inputs to soil and SOC content induced by a bioenergy plantation with poplar SRC on former cropland and pasture. We previously showed that after three years of coppice plantation below-ground carbon inputs, including roots and mycorrhizae, were more effective than above-ground litter in increasing SOC stocks (Berhongaray et al. 2019). Soil carbon dynamics, however, are slow and complex and questions remained regarding the long-term SOC dynamics in the bioenergy plantation and the underlying mechanisms. How do SOC stocks change in poplar plantations at a decadal scale? What are the relative contributions of different below-ground carbon input pathways (roots, mycorrhizae, dissolved organic matter and DOC) to this process? To address these questions we conducted a soil sampling campaign and an in-growth cores experiment with a distinct ^13^C soil signature; different mesh sizes enabled us to study the dominant carbon input pathways and their effect on new and native SOC stocks.

This study builds on earlier research conducted at the Lochristi plantation in Belgium where SOC changes were first assessed four years after plantation establishment (2010–2014), showing notable increases in SOC in croplands but minimal changes in pastures (Berhongaray et al. 2017). A subsequent study examined carbon input pathways after three years (2011–2013) using δ^13^C-labeled in-growth cores, highlighting the dominant role of root inputs in SOC accrual (Berhongaray et al. 2019). The results suggested that below-ground inputs – particularly from roots and mycorrhizae – were more effective than above-ground litter in increasing SOC. However, soil carbon dynamics are slow and complex, and long-term effects of bioenergy plantations on SOC remain insufficiently explored, particularly at the decadal scale.

The main objective of this study was to assess the long-term effects (over 12 years) of an SRC poplar bioenergy plantation on SOC stocks and carbon input pathways in former pasture and cropland. We conducted repeated soil coring (2010 and 2023) and a 12-year in-growth core experiment (2011–2022) using a distinct δ^13^C soil signature and different mesh sizes to differentiate the contribution of roots, mycorrhizae, and DOC to new SOC formation. Specifically, we tested the following hypotheses:

1. The poplar bioenergy crop increases SOC stocks in former cropland (with low initial SOC) but not in former pasture (with higher initial SOC).
2. Mycorrhizal fungi inputs are more important for SOC accrual than fine root inputs.
3. The presence of roots accelerates the mineralisation of native soil organic matter, leading to SOC loss, but this is outweighed by the formation of new SOC.

## Materials and methods

### Experimental site

**Fig. 1:**
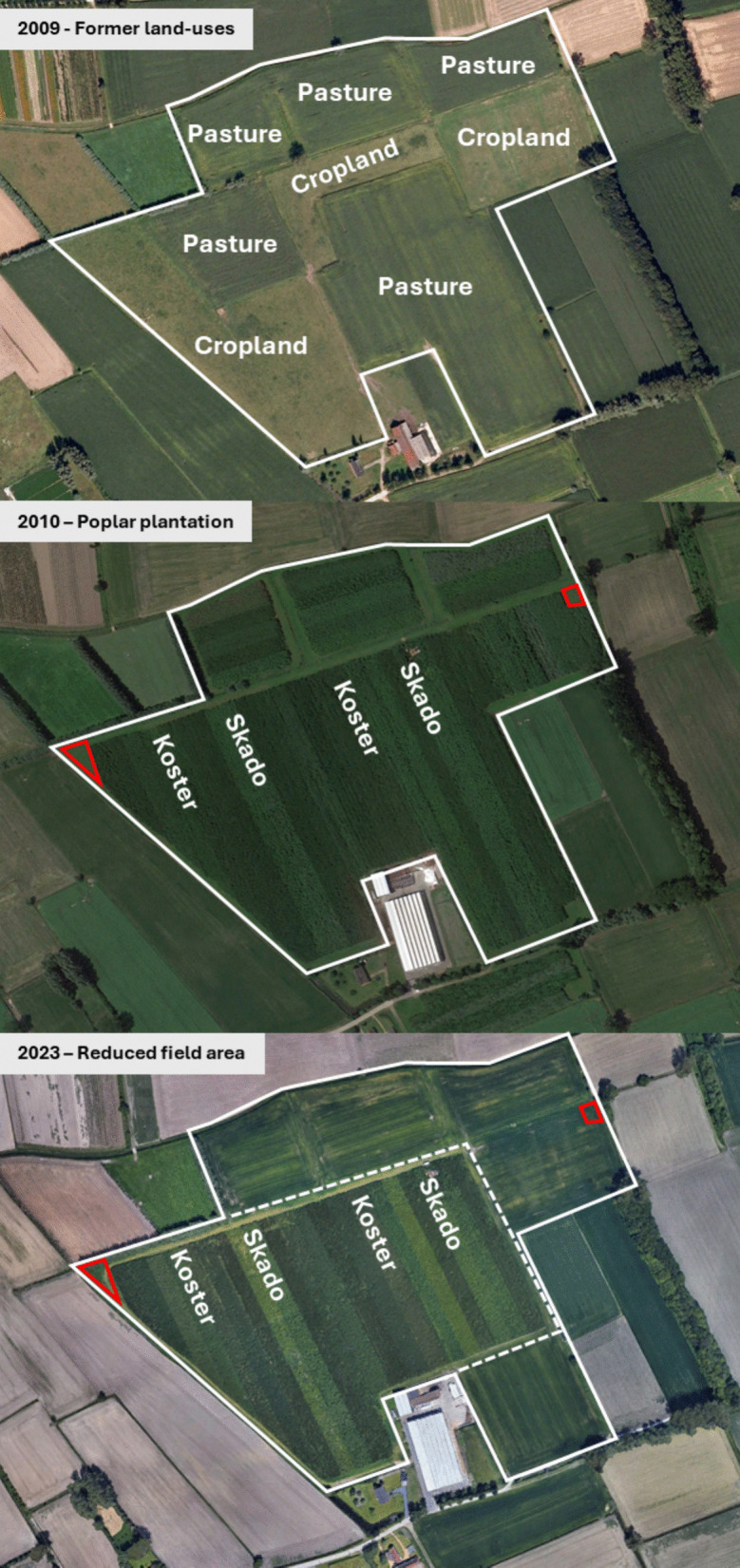
Aerial view of the experimental field showing: (top) the distribution of former land uses in 2009 (pasture or cropland); (middle) the field layout in 2010 after poplar plantation with the location of the two genotypes (Skado and Koster); and (bottom) the reduced field area in 2023. White lines indicate field boundaries and labels denote former land use or genotype. Red squares mark unplanted reference areas under permanent pasture (Source: Google Earth)

The experimental field site was located in Lochristi, Belgium (51°6′44"N—3°51′02"E) and consisted of a high-density plantation of large monospecific and mono-genotypic blocks of poplar (*Populus* spp.). Lochristi is located 11 km from Ghent in the province of East-Flanders at an altitude of 6.25 m above sea level with a flat topography. The long-term average annual temperature at the site is 9.5 ºC and the average annual precipitation is 726 mm (Royal Meteorological Institute of Belgium). The site is located in a flat, low-lying area close to sea level with naturally slow drainage due to minimal topographic relief. Although the soil has a sandy texture and good infiltration capacity, water percolation is limited by the shallow regional water table and slow lateral flow. The soil type according to the World Reference Base is Dystric Protic Arenosol (IUSS Working Group WRB 2015). The total area of the site was 14.5 ha in 2010 and was reduced to 8.5 ha in 2023 due to a reclamation for agricultural purposes by the land owner (Fig. 1). The two former land-use types of the site were (i) cropland (monoculture corn with regular nitrogen fertilization at a rate of 200–300 kg ha^−1^ y^−1^ as liquid animal manure and chemical fertilizers, alternating with winter crops including ryegrass and wheat), and (ii) extensively grazed pasture. A detailed soil analysis was carried out in March 2010 (Table 1), prior to land preparation and planting Soil nutrient concentrations (P, K, Ca, Mg, Na) were determined on composite samples of the soil layers at 0–30 cm and 30–60 cm depth, by the Soil Service of Belgium, using the ammonium lactate–acetic acid extraction method (pH 3.75, AL extract) to assess plant-available fractions (Amery et al. 2021). Values are expressed in mg/100 g dry soil, and reported as mean ± SE and range. The analysis characterized the soil type as a sandy texture. In the upper soil layer, C and N concentrations were significantly (*p* < 0.05) lower in cropland as compared with pasture and decreased exponentially with depth in both former land-use types. More details on the sampling and statistical analysis can be found in Broeckx et al. (2012).

**Table 1:** Soil pH, nutrient mass fractions and particle size distribution of the soil layers at 0–30 cm and 30–60 cm depth. Nutrient concentrations represent plant-available fractions, extracted using ammonium lactate-acetate (AL) method by the Soil Service of Belgium. Values are expressed in mg/100 g dry soil and shown as mean ± SE (range), for both land use types pasture and cropland over the total land area. Particle size distribution indicates that the soil has a texture of loamy sand

	Cropland		Pasture	
	0–30 cm	30–60 cm	0–30 cm	30–60 cm
pH—KCl	5.5 ± 0.1	5.9 ± 0.3	5.1 ± 0.2	5.6 ± 0.2
P [mg/100 g soil]	28.4 ± 4.3	8.6 ± 2.2	18.6 ± 2.5	6.4 ± 2.4
K [mg/100 g soil]	17.2 ± 2.9	12.0 ± 3.2	10.2 ± 2.1	5.8 ± 1.1
Mg [mg/100 g soil]	12.8 ± 0.9	13.7 ± 1.6	14.0 ± 1.0	11.1 ± 1.3
Ca [mg/100 g soil]	111.4 ± 7.6	249.8 ± 147.1	103.1 ± 12.4	99.0 ± 10.9
Na [mg/100 g soil]	1.5 ± 0.2	1.9 ± 0.2	1.5 ± 0.1	1.3 ± 0.1
Clay < 2 µm	10.1 ± 1.4	11.5 ± 1.0	11.5 ± 0.4	10.1 ± 1.2
Silt 2—10 µm	2.4 ± 1.9	1.5 ± 0.1	0.6 ± 0.2	2.6 ± 1.3
Silt 10—20 µm	0.5 ± 0.2	1.1 ± 0.2	0.4 ± 0.1	0.9 ± 0.1
Silt 20—50 µm	0.9 ± 0.2	1.6 ± 0.2	0.9 ± 0.1	2.0 ± 0.2
Sand > 50 µm	86.2 ± 1.2	84.3 ± 0.9	86.6 ± 0.5	84.5 ± 0.4

*KCl* potassium chloride; *P* phosphor; *K* potassium; *Mg* magnesium; *Ca* calcium; *Na* sodium

Soil preparation was performed by intensive ploughing (40–70 cm depth), tilling and a pre-emergent herbicide treatment. These operations were uniformly applied across the entire field, regardless of former land use, and served to homogenize soil conditions prior to planting. Although croplands had a history of regular tillage, pastures did not; thus, the intervention acted as a reset of soil structure and vertical SOC distribution. This allowed for a standardized starting point from which the effects of land-use legacy and poplar inputs could be evaluated under comparable conditions. After soil preparation, 25 cm long dormant unrooted cuttings from 12 poplar genotypes were planted in April 2010 in mono-genotypic blocks in a double-row planting scheme with a commercial leek planter (Broeckx et al. 2012).

Due to the high labor cost and to limit the variability caused by different species and genotypes, only two genotypes were assessed for detailed soil C balance assessment for the current study: i.e. Koster (*Populus deltoides* Marsh x *P. nigra* L.) and Skado (*P. trichocarpa* Hook. X *P. maximowiczii* Henry). Both genotypes were chosen because they are genetically and phenotypically contrasting and they represented the range of productivity values for the entire plantation (see Broeckx et al. 2012 and (Verlinden et al. 2015) for more details on the productivity of the genotypes).

In the double-row planting scheme, the distance between the narrow rows was 75 cm, while the distance between the wide rows was 150 cm. The distance between trees within a row was 110 cm, yielding an overall density of 8000 trees per ha. The total length of individual rows ranged from 45 m up to more than 325 m. Manual and chemical weed control was applied during the first and the second years. During the first months after planting intensive weed control – mechanical, chemical and manual – was applied to decrease competition for light and nutrients. With the exception of glyphosate, none of the herbicides used specify poplar as an approved crop species for the use of these chemicals. Herbicides that have proven effective in the establishment of poplar plantations in experimental trials were chosen based on the weed species present in the field (personal communication F. Goossens, ILVO). A full chronology with dates, target species, application areas, frequency, and any observed tree damage is provided in Supplementary Table S7. Neither fertilization nor irrigation was applied during the entire lifetime of the plantation. Two small portions of the field remained untouched with pasture as unplanted control plots for the determination of soil changes (Fig. 1). The plantation was managed in five two- and one three-year rotations. At the end of each rotation the entire crop was machine coppiced and allowed to resprout.

### Soil carbon stock

Soil organic carbon (SOC) stock changes were assessed by repeated soil coring in March 2010 (at plantation establishment), and March 2023 (after the sixth rotation). A stratified-random sampling was performed at 110 locations in March 2010 of which 40 were located within the two selected genotypes Skado and Koster, and eight were located within the reference areas under permanent pasture (Fig. 1). In March 2023 locations were re-sampled (Koster: cropland = 5; pasture = 7; Skado: cropland = 4; pasture = 3), and within each land-use type half in each of the two row spacings (in wide rows and in narrow rows between poplar stools). In both March 2010 and March 2023, soil samples were collected down to a depth of 90 cm, in 15 cm intervals. To reach the target depth zones an Edelman auger was used, and an undisturbed sample was then taken from each layer using a Kopecky ring (5 cm height, Ø 53 mm, 100 cm^3^ volume; Eijkelkamp Agrisearch Equipment, The Netherlands) to determine bulk density (BD). From each ring, a subsample was extracted to analyse carbon and nitrogen content with an NC element analyser (see below). Given that only 5 cm of each 15 cm layer was sampled directly SOC content for each full interval was estimated using interpolation through smoothing spline functions (Berhongaray et al. 2013). While this method introduces some uncertainty, it provides a practical and robust approach for reconstructing SOC profiles in field conditions. The soil had a low initial pH ranging between 5.1 and 5.9 (Table 1) and no liming was applied during the 12 years of the experiment making the presence of carbonates in the soil unlikely. Hence, SOC and SON mass fractions were determined with an element analyser (NC-2100, Carlo Erba Instruments, Italy). From the SOC mass fractions and the BD, the C pool per 15 cm depth interval was calculated, and cumulated over 90 cm. SOC data were transformed to equivalent soil mass to account for differences in BD induced by the previous land-use, poplar genotype and row spacing. The estimations of SOC stock at equivalent soil mass were performed for soil masses of 330 and 1070 kg m^−2^ using the spline functions. For the spline functions, the soil mass was used as the independent variable and SOC as the dependent variable. Interpolations were made by adding or by removing a portion of the soil to reach the desired soil mass assuming that transitions between soil layers were smooth and continuous, which at a soil depth of around 90 cm was a reasonable assumption.

### Soil carbon dynamics

Mineralisation of soil organic matter and inputs were studied using in-growth cores with different mesh sizes filled with soil from a C_4_ grassland (further referred to as C_4_ soil) with a distinct ^13^C signature collected in a short grass steppe in Colorado (USA), and applying an isotopic mixing model to quantify new SOC formation and C₄-derived SOC losses (described in detail in (Berhongaray et al. 2019). In-growth cores were installed in 2010. A known volume container was filled with oven-dried soil and weighed to determine the mass-to-volume ratio. Based on this ratio we calculated the amount of soil needed to fill each core to a given depth (e.g., 10 cm), and then compacted the soil until the target depth and mass were achieved. This approach allowed us to standardize BD among cores and treatments despite the absence of in situ measurements of core compaction. During the in-growth core installation in the soil of the plantation seven samples were randomly taken from the C_4_ soil, representing the initial C_4_ soil (Soil 0). The samples were oven-dried at 40° C in the laboratory and stored in plastic bags until analysis. Additionally, we manipulated mesh sizes (see below) to assess the individual versus combined contributions to SOC of the different plant inputs.

**Fig. 2:**
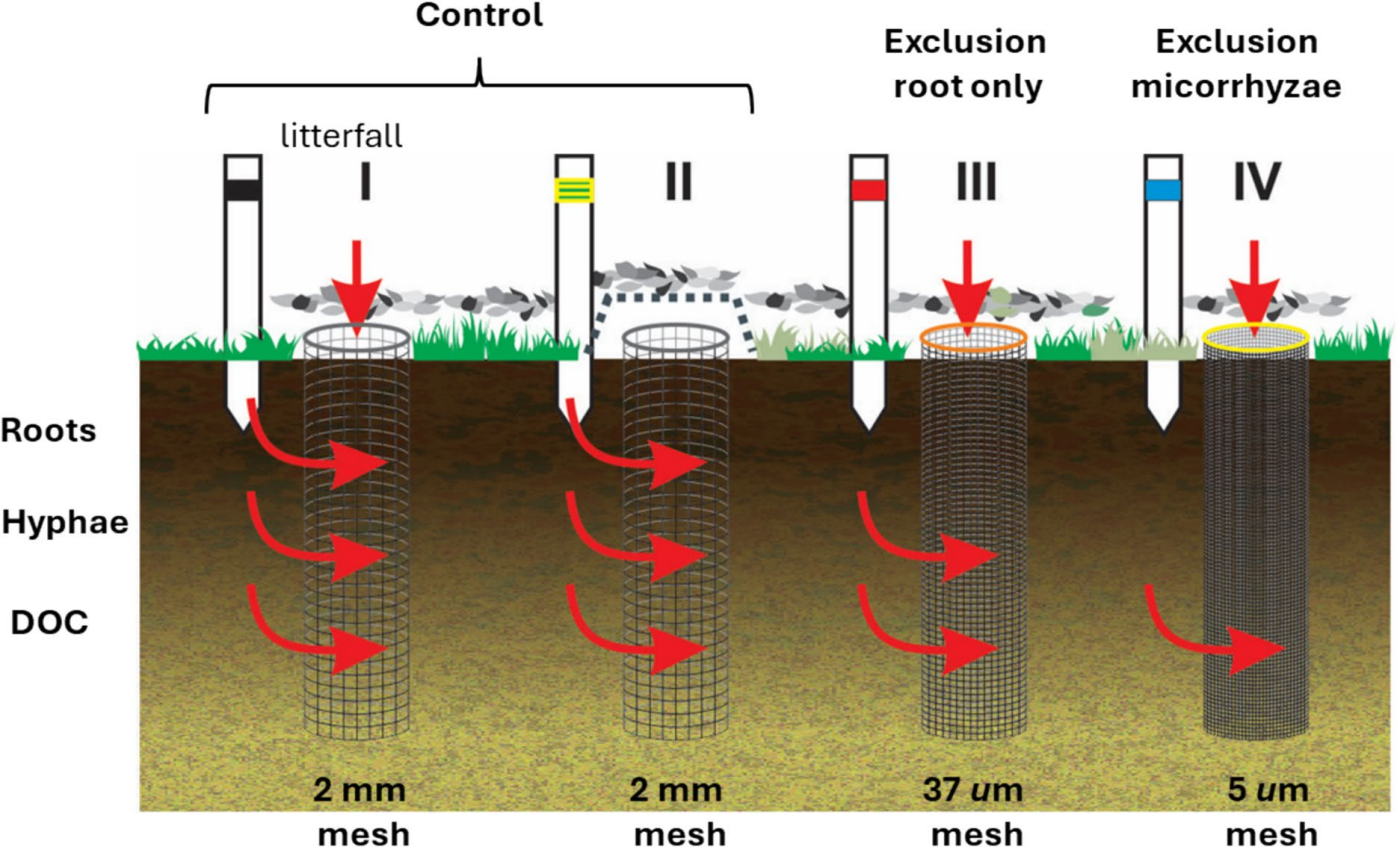
Schematic representation of the four experimental treatments used to assess carbon input pathways in the soil. Treatment I allows inputs from roots, mycorrhizal hyphae, and dissolved organic carbon (DOC). Treatment II excludes above-ground litter inputs but allows below-ground inputs (roots, hyphae, and DOC). Treatment III excludes roots but allows hyphal and DOC inputs. Treatment IV excludes all below-ground inputs, capturing only above-ground litter contributions

In the original experiment (established in 2010) four treatments were implemented using mesh sizes of 2 mm, 37 µm, and 5 µm to selectively exclude above-ground inputs, roots, and mycorrhizal hyphae. A schematic representation of the treatments and mesh configurations is shown in Fig. 2. The four treatments were:

Treatment I (all inputs): a mesh size of 2 mm allowed inputs from fine roots and mycorrhizal mycelia. The opening on the top also allowed inputs from above-ground (leaf and wood litter).

Treatment II (exclusion of above-ground input): the same mesh size (2 mm) as in Treatment I, but a 10 mm mesh net on top of the in-growth core and the periodic removal of above-ground litter from the top prohibited above-ground inputs. However, due to practical challenges over the 12 years of the experiment, above-ground inputs could not be consistently excluded in this treatment. As a result, Treatments I and II were merged into a single treatment for the analysis, referred to as "Control." This combined treatment represents conditions where inputs from fine roots, mycorrhizal mycelia, and above-ground sources were present.

Treatment III (Exclusion root only): an intermediate mesh size (37 µm) excluded the access of roots, but allowed the in-growth of mycorrhizal mycelia. Above-ground inputs were also allowed.

Treatment IV (Exclusion mycorrhizae): the smallest mesh size (5 µm) excluded the in-growth of roots and mycorrhizal mycelia, allowing only the inputs of above-ground litter and DOC from surrounding soil.

These three in-growth treatments were applied in the two genetically and phenotypically contrasting poplar genotypes (i.e. Koster and Skado). The in-growth cores were placed in both of the two former land uses (pasture and cropland), for a period of two and three years and reported previously in Berhongaray et al. (2019)), and a period of 12 years (2011–2022; 12 growing seasons) reported in the current research. The in-growth cores were replicated five times per genotype, per former land use, per treatment and per sampling campaign, yielding a total of 240 in-growth cores. See the Supplementary Material for further details on coppice rotations.

### Sampling and sample manipulation

A first and a second sampling campaign was carried out in 2012 (after two growing seasons; 80 cores), and 2013 (after three growing seasons; 80 cores) in both of the two former land uses (pasture and cropland). The results of those samplings were described in detail and were published earlier (Berhongaray et al. 2019). In the current study we carried out a third sampling in November 2022, when 60 out of 80 in-growth cores left were retrieved and extracted from the soil (Table 2). Although the exact position of the in-growth cores was recorded during installation with a GPS device, the localization of the in-growth cores was a difficult task, as some of the in-growth cores were slightly buried underneath the soil surface. In November 2022 soil samples were taken in the middle of each in-growth core with a BD corer (5 cm diameter and 5 cm length; Eijkelkamp Agrisearch equipment, The Netherlands), at 5 cm increments from the soil surface to 60 cm depth. To obtain a good representation of SOC formation at the top, middle and deeper soil horizons, while avoiding unmanageable sample numbers, the 5–10 cm, 15–20 cm, 25–30 cm and 50–55 cm samples were chemically analysed, while the rest of the soil samples were discarded. The first sample on top (0–5 cm) as well as the last sample from the bottom (55–60 cm) of the C_4_ soil sample were discarded to avoid contamination from the surrounding C_3_ soil. In total 215 soil samples were analysed from the November 2022 sampling. The sampling in November 2022 differed slightly from the previous two samplings in 2012 and 2013 when the 0–5 cm, 10–15 cm and 30–35 cm were sampled and a total of 480 samples were obtained (Berhongaray et al. 2019). Due to differences in sampling design and depth resolution, the earlier datasets were not included in the present analysis. Instead, the 2022 sampling was treated as an independent study, allowing for a consistent and internally comparable assessment of carbon inputs and stocks across treatments.

**Table 2:** Number of in-growth cores recovered from each combination of genotype and former land use

Genotype	Former land use	Number of in-growth-cores
Koster	Cropland	16
	Pasture	16
Skado	Cropland	20
	Pasture	8
TOTAL		60

The samples were put in plastic bags and kept in a freezer until processing. During processing, each soil sample was thawed and weighed to determine fresh weight (FW). Roots were manually removed from the fresh sample, oven-dried at 70 °C, and weighed to determine root dry biomass. The remaining soil was then dried at 45 °C for seven days and weighed again to calculate water content. The dry soil was sieved through a 2 mm mesh to separate remaining coarse fragments (roots and stones) that were weighed. No stones were found in the samples. BD was calculated by subtracting from the total sample weight the tare weight of the sampling cylinder, the dry mass of roots, and the mass of coarse fragments (Jurgensen et al. 2017). The resulting value was divided by the known volume of the cylinder to obtain the BD of the fine earth fraction. The sieved fine earth was then pulverized in a mill (Retsch model ZM 200, Germany). Soil samples from 2022 were sent out to the Isotope Bioscience Laboratory (ISOFYS) at Ghent University (Ghent, Belgium) for the determination of C% and δ^13^C by an Elemental Analyser-Isotope Ratio Mass Spectrometer (EA-IRMS model NA1500, Carlo Erba, Italy coupled to a VG Isochrom continuous flow IRMS, Isoprime Inc., UK). Each C_4_ soil sample was acid fumigated to eliminate any carbonate (Harris et al. 2001) and analysed in duplicate. In case of major differences (more than 5%) between the two results, a third and eventually a fourth analytical replicate was performed. This procedure increased the confidence of the δ^13^C value of the sample. From the C mass fractions and the BD the SOC pool for each depth interval was calculated.

### Partitioning of the poplar derived soil organic carbon

New SOC originating from above- and below-ground inputs was determined using the two end-member mixing model (Balesdent et al. 1988). The fraction (*f*) of SOC coming from the trees was calculated as:

1 $$f= \frac{{\updelta 13\mathrm{C}}_{Soil sample}-{\updelta 13\mathrm{C}}_{Soil 0}}{{\updelta 13\mathrm{C}}_{Plant}-{\updelta 13\mathrm{C}}_{Soil 0}}$$

where δ^13^C_Soil sample_ = δ^13^C of the SOC at the sampling time, δ^13^C_Soil0_ = average δ^13^C of the SOC in the original C_4_ soil, and δ^13^C_Plant_ = average δ^13^C of the poplar C input. Average δ^13^C_Plant_ was calculated considering the relative contribution of the different sources (leaves, wood and roots) to the total litter input for each treatment and soil depth. For example, treatment I at 5–10 cm of soil depth received inputs from above (leaf and wood litter) and from roots, while the deeper soil depths (15–20 cm 25–30 cm and 50–55 cm) received inputs from roots only. Consequently the average δ^13^C_Plant_ for the top soil was calculated by multipliying the δ^13^C from leaves, wood and root tissues, weighted by their relative contribution to the total C input, i.e. 70%, 20% and 10% respectively. On the other hand, for the deeper soil layers only the root δ^13^C was used. The new SOC ($${soil C}_{new})$$, or the poplar-derived C in each sample was determined from the total SOC pool $${(soil C}_{total}$$) and *f* as follows:

2 $${Soil C}_{new}={Soil C}_{total}*f$$

Inversely, the remaining C₄-derived SOCwas determined as follows

3 $${Soil C}_{4-derived}={Soil C}_{total}*(1-f)$$

### Statistical analyses

The *f* and the absolute amounts of C₄-derived SOCand poplar-derived C in the soil were calculated for each of the 48 experimental factor combinations (genotypes, former land uses, input treatments, and soil depths). To test for treatment effects, we conducted a factorial analysis of variance (ANOVA) with genotype, former land use, input treatment, and soil depth as fixed factors. More specifically, C and N concentrations were analysed using repeated measures ANOVA with year (2010 vs. 2023) as the within-subject factor and land use as a between-subject factor. New poplar-derived carbon stocks (from the in-growth cores) were analysed using factorial ANOVA, testing for main effects and interactions among genotype, land use, input treatment, and depth. Differences between means were assessed using Fisher’s least significant difference (LSD) test at p ≤ 0.05. Simple linear regressions and Pearson correlation analyses were conducted to explore relationships among variables such as root-derived carbon input and SOC change. All analyses were performed in InfoStat (Di Rienzo et al. 2011). Assumptions of normality and homoscedasticity were checked graphically and with Shapiro–Wilk and Levene’s tests, respectively. Further methodological details and descriptive statistics are provided in the Supplementary Material.

## Results

### Soil carbon and nitrogen concentrations, and BD

**Fig. 3:**
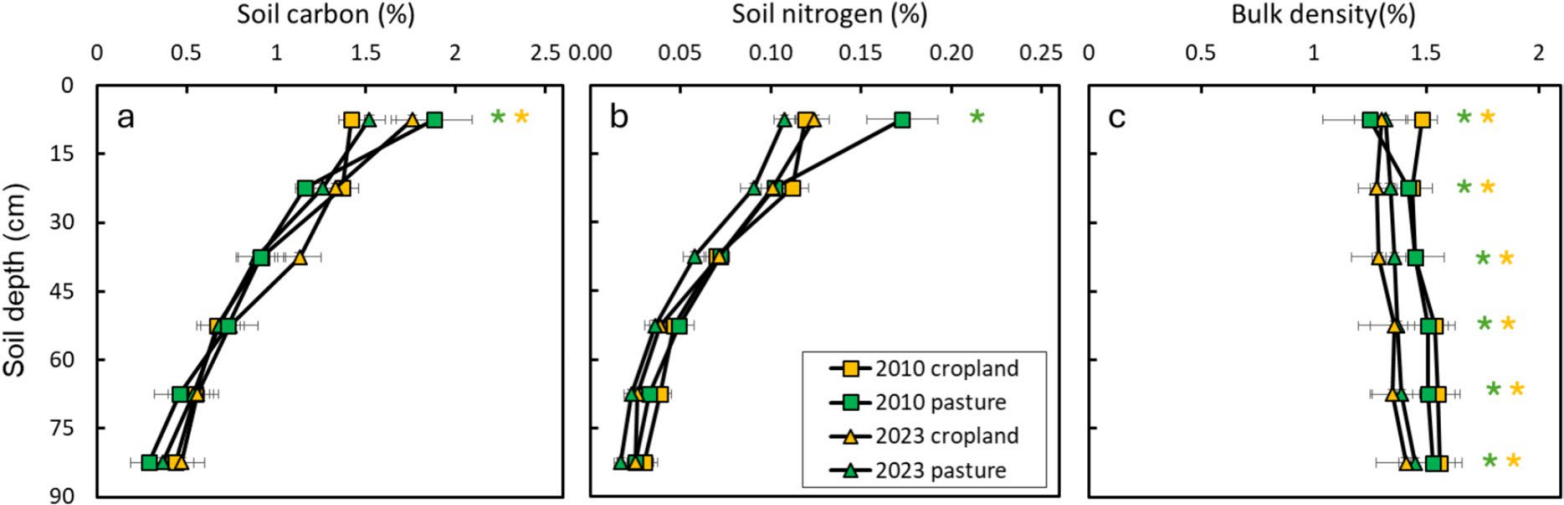
Distribution of soil organic carbon concentration (**a**), nitrogen concentration (**b**) and bulk density (**c**) with soil depth sampled in March 2010 in former cropland and pasture lands and in 2023 after six short-rotation coppice cycles. Asterisks represent significant statistical differences (*p* < 0.05) between the 2010 (squared symbols) and 2023 (triangle symbols) samplings green symbols = pasture, yellow symbols = cropland. Horizontal bars represent standard error of the mean

The pattern of soil C concentration over time differed from the one in soil N concentration (Figs. 3a and b). The evolution of SOC and N concentrations between 2010 and 2023 varied depending on former land use. In croplands only the top 10 cm increased in SOC 12 years after planting (Fig. 3a). Conversely, in pastures there was a decrease in C concentrations although less pronounced. Soil N concentrations (Fig. 3b), in contrast, declined with time in former pasture, but not in former cropland. BD decreased in both land-use types and across all soil depths. On average across the 0–90 cm profile, BD decreased by 5% in croplands and by 12% in pastures.

**Fig. 4:**
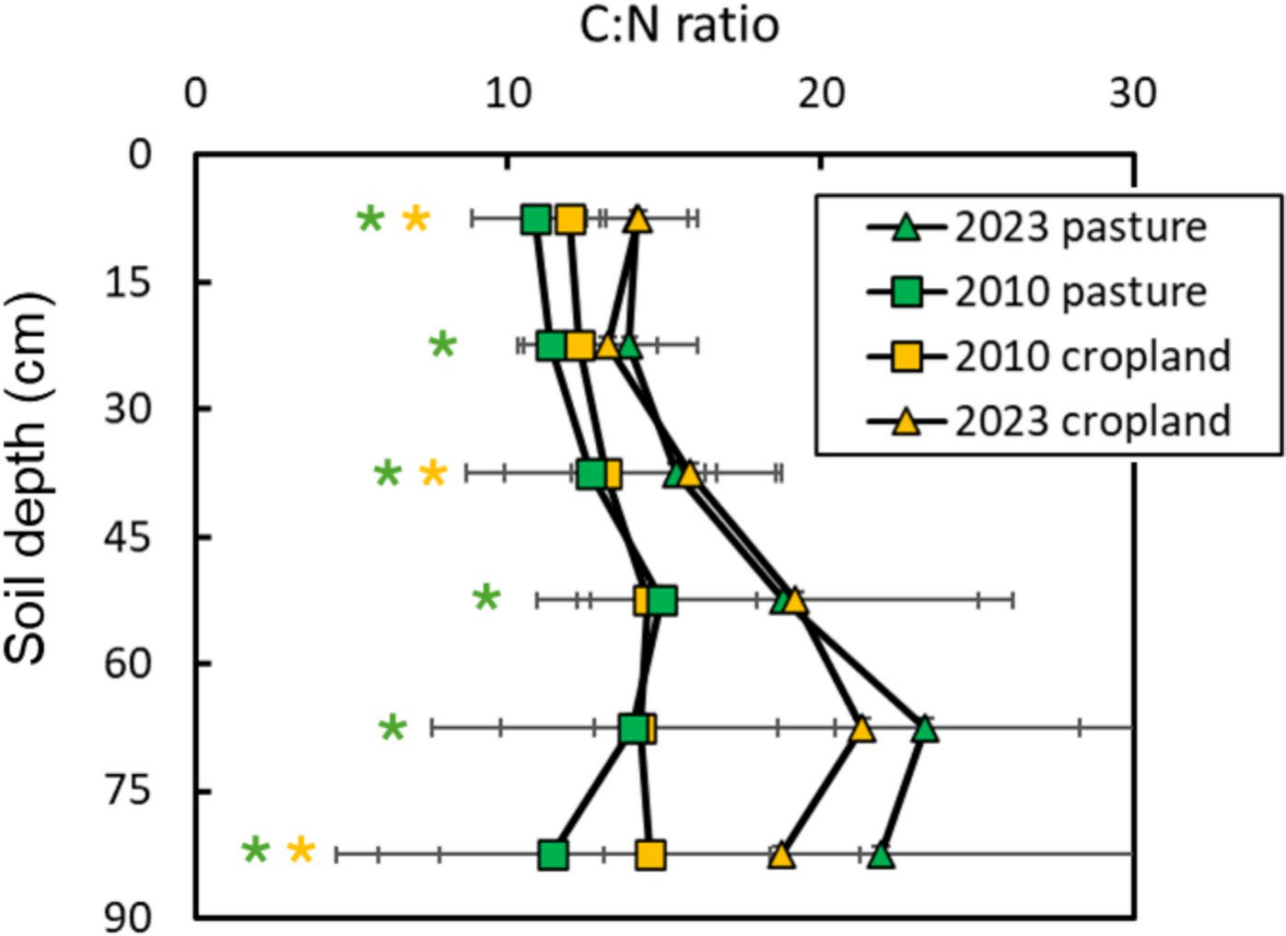
Distribution of carbon/nitrogen (C:N) ratio with soil depth sampled in March 2010 in former cropland and pasture lands and in 2023 after six cycles of SRC. Asterisks represent statistical differences between 2010 and 2023 sampling (*p* < 0.05), green symbols = pasture, yelow symbols = cropland. Horizontal bars represent standard error of the mean

As a result of the contrasting changes in soil C and N, the carbon/nitrogen (C:N) ratio increased in both former land uses at all evaluated soil horizons, albeit more strongly in deeper soil layers (Fig. 4). In the surface layer, the C:N ratio was initially higher in croplands (12 ± 4.1) than in pastures (10.9 ± 3.2) in 2010, but ratios converged to a similar value (14 ± 7.0) in 2023. Across all the depths from 15 to 90 cm the C:N ratio increased from 2010 to 2023. The results thus show that the SRC systematically increased C:N ratio but that this increase was more pronounced in the deeper soil layers.

**Fig. 5:**
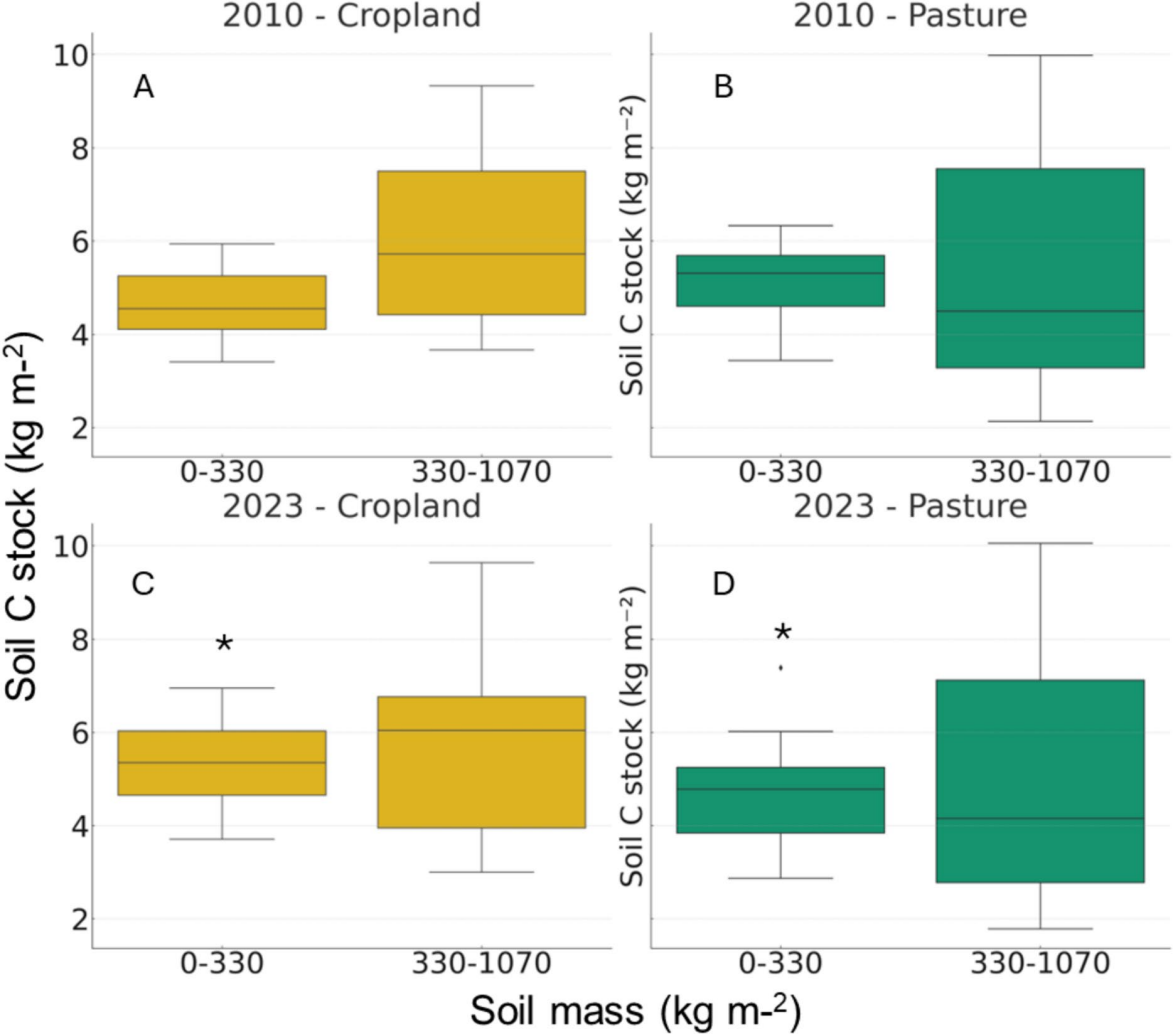
Soil carbon stocks at equivalent soil mass in former cropland (left panels **A** and **C**, yellow) and pasture lands (right panels **B** and **D**, green) in 2010 (upper panels **A** and **B**) and 2023 (lower panels **C** and **D**). Soil carbon stocks were calculated at an equivalent soil mass of 330 kg m⁻^2^ and 1070 kg m⁻.^2^, roughly corresponding to a depth of ~ 30 cm and ~ 90 cm respectively. The plots show the distribution of the measured values, including median, interquartile range, and potential outliers.. * Statistically significant differences between sampling years at *p* < 0.05

In the topsoil (0–15 cm), relative to the initial conditions in 2010 BD increased by 5% in former pastures and decreased by 13% in former croplands (Fig. 3c). Below 15 cm BD decreased in both former land uses by about 10%. When accounting for these changes in BD and expressing soil carbon stocks per unit equivalent soil mass, contrasting trends between former cropland and pasture were observed. While former cropland showed a significant increase in SOC stock with time, particularly in the upper soil layers, pastures declined over the study period (Fig. 5) although these changes were only significant in the surface layer.

**Fig. 6:**
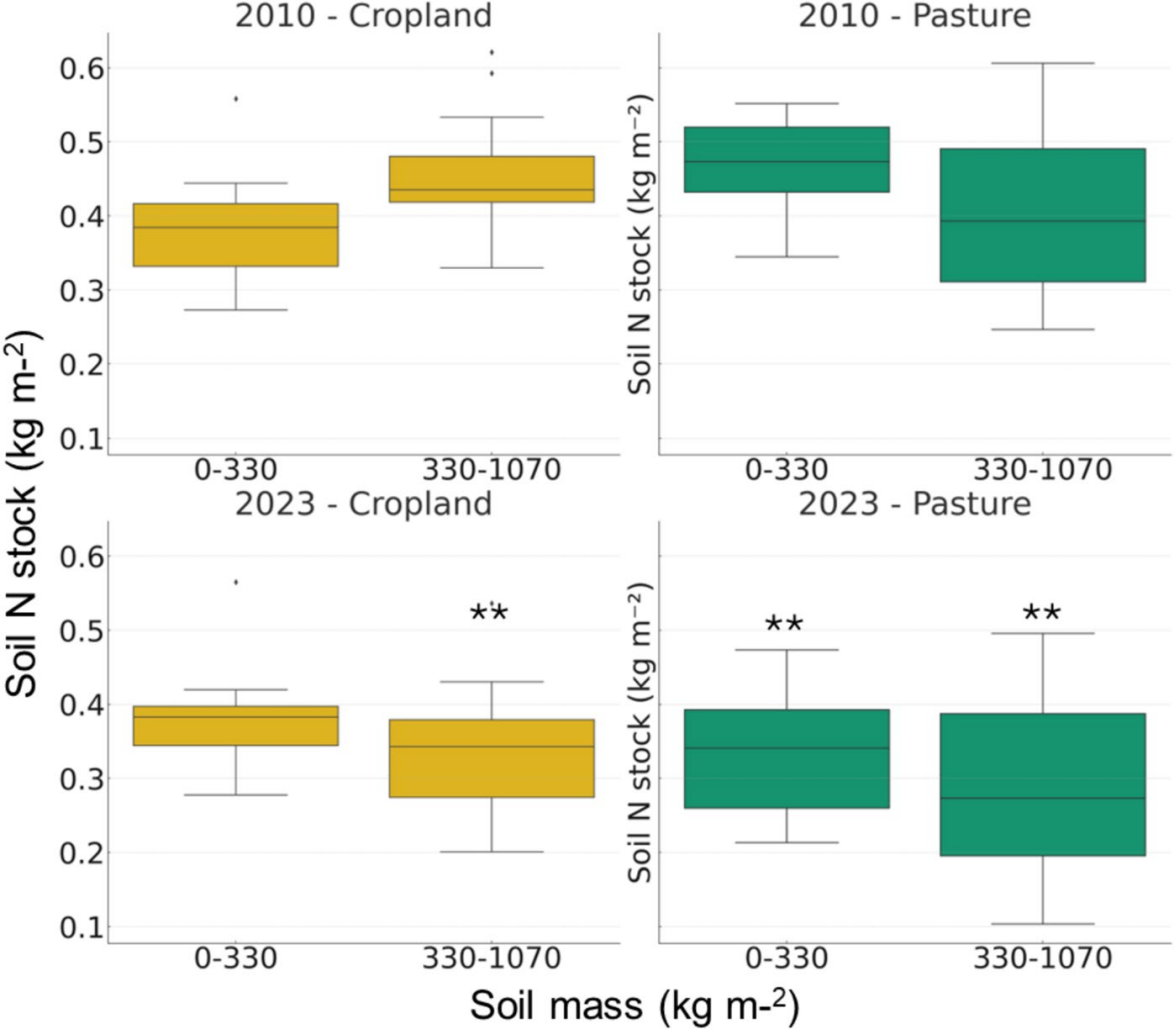
Soil nitrogen stocks at equivalent soil mass in former cropland (right panels **A** and **C**, yellow) and pasture lands (left panels **B** and **D**, green) in 2010 (upper panels **A** and **B**) and 2023 (lower panels **C** and **D**). Soil nitrogen stocks were calculated at an equivalent soil mass of 330 kg m⁻^2^ and 1070 kg m⁻.^2^, roughly corresponding to a depth of ~ 30 cm and ~ 90 cm respectively. Error bars represent standard errors. The plots show the distribution of the measured values, including median, interquartile range, and potential outliers. ** statistically significant differences between sampling years at *p* < 0.01

Changes in soil N stock with time were more pronounced than those of C (Fig. 6). In the surface layers former pasture experienced a 30% decrease in N stocks, while former cropland did not change. In the deeper soil layers both former cropland and pasture showed reductions in soil N stock of 25% and 30%, respectively.

### Soil carbon dynamics

**Fig. 7:**
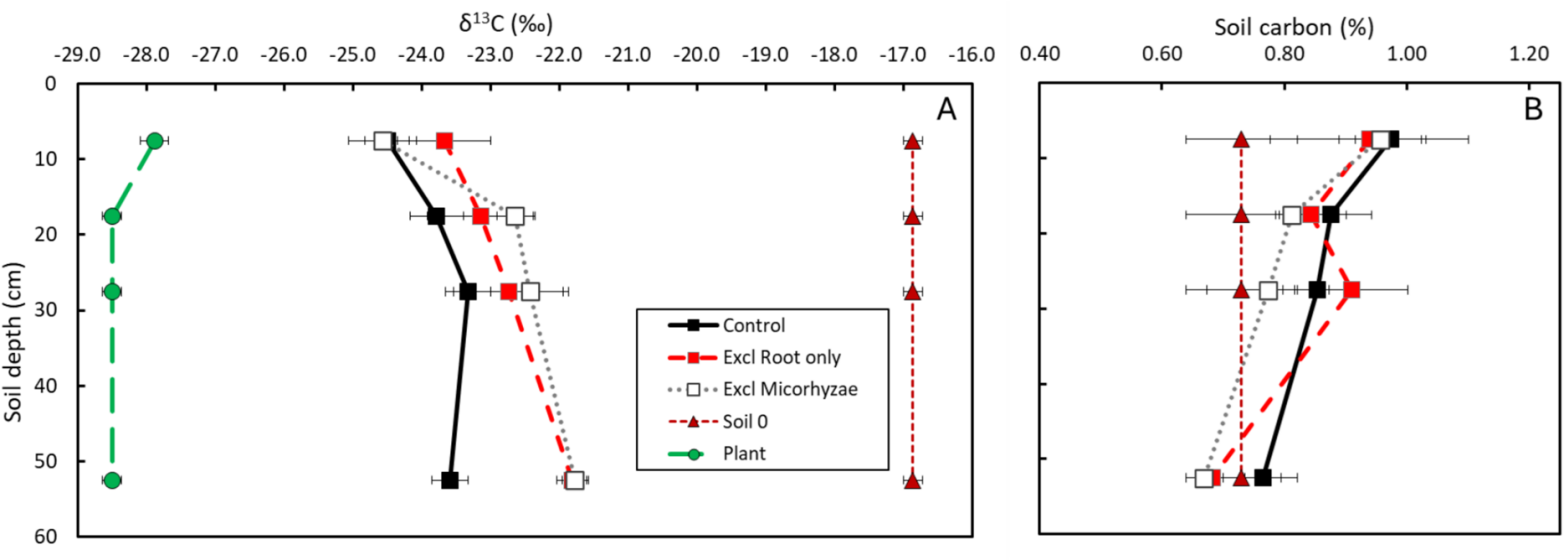
Average plant δ^13^C (in ‰) as well as initial and final – after 12 years – soil δ^13^C (in ‰; left panel **A**) and soil C concentration (in %; right panel **B**) across genotypes and land uses for each treatment at different soil depths. Plant δ^13^C (green circles) was calculated considering the relative contribution of the different sources (leaves, wood and roots) to the total litter input. Soil 0 (brown triangles) represents the initial C_4_ soil δ^13^C and C %. In-growth core treatments are represented with squares: Control treatment (Treatments I and II) in black squares, exclusion root only (Treatment III) in red squares and exclusion mycorrhizae (Treatment IV) in white squares. Horizontal bars represent standard error of the mean

After 12 years the soil C concentrations in the in-growth cores had increased, although this increase declined with depth (Fig. 7; right panel). The δ^13^C of the soil in the in-growth cores was substantially reduced relative to the original soil (pure C_4_ soil organic matter), reflecting the incorporation of organic matter derived from C_3_ poplar plants, i.e., the poplars (Fig. 7; left panel). The reduction in δ^13^C values was highest in the top layers, indicating larger poplar carbon inputs in the top soil.. The decrease in soil δ^13^C values over time (from 2010 to 2022) was observed across all treatments, genotypes (Skado, Koster), and former land uses (cropland, pasture) (Supplementary Material Figure SM5). In most cases the exclusion root only treatment (Treatment III, red squares, Fig. 7) showed a smaller decrease in δ^13^C compared to root inclusion treatment (combined Treatments I and II, black squares; Fig. 7). This indicates that the presence of roots significantly contributed to the incorporation of poplar derived C into the soil. The exclusion mycorrhizae treatment (Treatment IV, white squares, Fig. 7) often showed a similar or slightly slower decline in δ^13^C compared to the root treatment. This suggests that mycorrhizal fungi also played a role in the C_3_ carbon incorporation but their impact was less pronounced or depended on other factors.

**Fig. 8:**
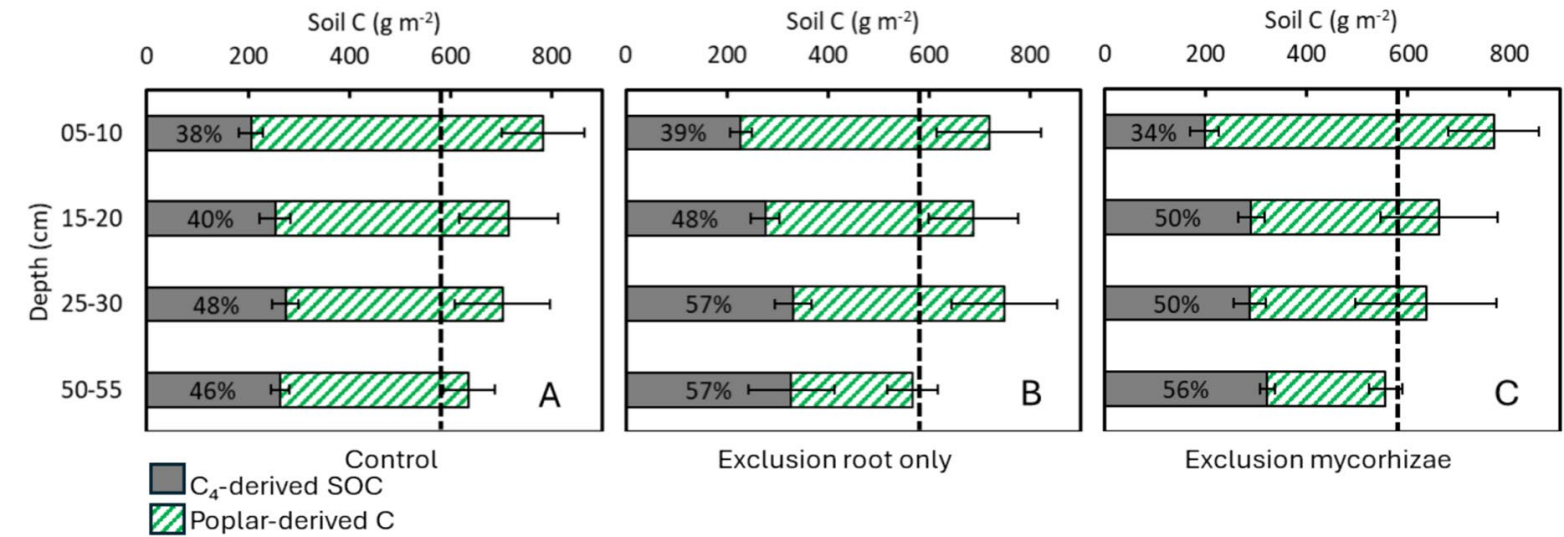
Soil carbon stock (g m⁻^2^) inside the in-growth bags at different soil depth intervals (0–5, 15–20, 25–30, and 50–55 cm) and treatments. C₄-derived SOC is the portion of soil carbon remaining from the original C_4_ soil before the experiment establishment; Poplar-derived C is the portion of soil C derived from the poplar trees. Treatments: Control: in-growth cores allowing full root and mycorrhizal access (Treatment I and II); Exclusion root only (Treatment III): in-growth cores excluding roots but allowing mycorrhizal access; Exclusion mycorrhizae (Treatment IV): In-growth cores excluding both roots and mycorrhizae. The dashed line represents the initial soil C stock at the beginning of the experiment. The percentage in the grey bars represents the remaining C₄-derived SOCfrom the initial soil C stock. The bars represent standard error of the mean

Integrating the total SOC stocks across the 0–60 cm soil profile of the in-growth cores (Fig. 8) revealed that the initial total SOC stock was approximately 6914 g C m^−2^. A decrease in C₄-derived SOCwas observed across treatments, with a loss of 3850 g C m⁻^2^ in the control treatment, suggesting changes in SOC composition over time. Proportionally, that decrease was higher in the upper layer than in the deeper layers. The final SOC was a combination of new poplar-derived C and C₄-derived SOC. The exclusion of mycorrhizae led to final SOC to about 7548 g C m^−2^, indicating that above-ground litter and DOC inputs alone contributed to a net increase of 633 g C m^−2^. Further inclusion of mycorrhizae, while excluding roots, increased the total SOC to approximately 7888 g C m^−2^ suggesting an additional net 340 g C m^−2^ attributed to mycorrhizae. Finally, the presence of all inputs, including roots, in the control treatment resulted in the highest total SOC of about 8281 g C m^−2^, indicating a further 393 g C m^−2^ net increase attributed to root inputs.

Across all treatments, the proportion of poplar derived C (Fig. 8; striped) decreased with soil depth, while the proportion of remaining C_4_ carbon (grey bars) increased. New carbon from the poplar plantation was thus incorporated into the soil faster in the topsoil than in deeper layers. The "Exclusion mycorrhizae", treatment allowing DOC + surface litter, reported 4100 g C m^−2^ amount of poplar-derived carbon (0–60 cm). Comparing "Exclusion root only" and "Exclusion mycorrhizae" we found 152 g C m^−2^ extra poplar-derived carbon in the former (0–60 cm). Comparing "Exclusion root only" and “Control” we found that 972 g C m^−2^ derived from roots (0–60 cm). This indicates that while mycorrhizae facilitated C input, their contribution was less significant than that of roots themselves. The total SOC stock (represented by the total bar length on Fig. 8) generally increased across all treatments and in the top soil remained comparable to the initial stock (dashed line on Fig. 8). This confirms that the poplar plantation actively accrued carbon in the soil of the in-growth cores (hypothesis 3).

## Discussion

Our first hypothesis was that the bioenergy crop increased soil carbon stocks in former cropland, but not in former pasture. This hypothesis was based on the fact that croplands typically have lower SOC contents than pastures providing a higher potential for SOC accrual in croplands than in pastures. In croplands we indeed observed a slight increase in SOC while pastures exhibited a decline (0–15 cm), supporting the first hypothesis that poplar bioenergy crops enhance SOC stocks in former cropland but not in former pasture. Nonetheless, changes were much smaller than expected. It is worth noting that all plots, regardless of previous land use or poplar genotype, were subjected to the same deep ploughing treatment prior to planting. This uniform intervention minimized structural and chemical differences in the soil profile at the start of the experiment, reducing potential confounding effects on rooting depth or below-ground input patterns. Therefore, differences observed in SOC dynamics are likely attributable to legacy effects of land use and plant inputs, rather than to the ploughing operation itself. We speculate that the main reason for the weak (small) changes in SOC is that the soils, albeit frequently ploughed and above-ground biomass extracted for fodder during the long period before the coppice plantation was established, were quite rich in SOC for an agricultural system on loamy sand. The high SOC stock of the former land use is likely attributable to the high amount of manure and – in the cropland – of the winter cover crops that are ploughed in the soil prior to planting corn in spring. A more detailed assessment of the mineral-associated organic carbon fraction would have provided insights into the soil's carbon saturation potential (Stewart et al. 2009). In fact, the absence of a significant carbon saturation deficit could explain why additional carbon inputs did not result in substantial SOC accumulation over time. Other studies evaluating changes in SOC by poplar plantations either showed no changes (Mukherjee and Coleman 2020; Sun et al. 2015) or increases (Coleman et al. 2004; Sartori et al. 2007). A recent study of the conversion from conventional agriculture to poplar bioenergy plantations in four agricultural sites found no differences in SOC at a depth of 0–15 cm, although the study spanned only four years (Mukherjee and Coleman 2020). In a Canadian study initial SOC declined following plantation establishment with recovery to pre-establishment levels occurring within 6 to 10 years (Sun et al. 2015). Another 10-year-long chronosequence study in the USA noted a SOC accumulation of approximately 1.8% under irrigated and fertilized conditions (Sartori et al. 2007), hindering direct comparison with our poplar plantation that neither received fertilizers nor irrigation. Significant increases in SOC concentration were reported in poplar stands across 27 sites in the USA, albeit especially in areas with low inherent carbon prior to plantation (Coleman et al. 2004).

Our in-growth core experiment (0–60 cm; 12 years) showed that poplar-derived C entered the soil via all pathways but not equally. The treatment with only above-ground inputs plus DOC already accumulated substantial poplar-derived C; allowing mycorrhizal ingrowth added a small increment, while allowing roots yielded the largest additional increment and the highest final SOC stock. In other words, roots dominated the incremental formation of new SOC beyond surface/DOC inputs, even though all pathways mattered over time. These results put into perspective earlier short-term findings at the same site that emphasized below-ground over above-ground inputs (3-year window) (Berhongaray et al. 2019, 2017), by showing that over a decadal window above-ground/DOC make a large baseline contribution, with roots providing the biggest extra gain and mycorrhizae a smaller increment. This pattern aligns only partly with broader literature. Under elevated CO₂ with poplar, Godbold et al. (2006) reported mycorrhizal mycelium as the dominant C input route into SOM, exceeding leaves and fine roots, highlighting that fungal pathways can dominate under some conditions and timescales. Yet more recent syntheses show that mycorrhizal effects on SOC are complex—ranging from inputs via necromass to stimulation of decomposition and mineral-associated C dynamics—so their net contribution varies with soils, nutrients and plant allocation (Godbold et al. 2006; Hawkins et al. 2023). Our long-term field evidence therefore complements these views by showing strong root-driven accrual despite measurable mycorrhizal roles. Finally, a study across forest successions indicates that root production and microbe-derived inputs are especially important early on, with above-ground litter gaining relative importance later (Liu et al. 2022), this temporal shift is consistent with our site-level trajectory from early below-ground dominance to a larger long-term share from surface inputs.

The poplar plantation actively accrued SOC in a C-poor grassland soil inserted in the in-growth cores. To the best of our knowledge our 12-year study represents the longest study utilizing in-growth cores to understand changes in SOC. By demonstrating that SOC accumulation is feasible in carbon poor agricultural soils the current study reinforces the notion that bioenergy plantations established on marginal lands have high potential for providing environmentally and economically beneficial solutions without competing with food production (An et al. 2021). A more detailed analysis of the SOC changes showed that the mineralization of C₄-derived SOC was more pronounced in the upper soil layers than in deeper layers, likely reflecting higher microbial activity and greater input of fresh carbon near the surface. Across treatments, a larger loss of C₄-derived SOC was observed in the root inclusion treatment, suggesting that the presence of roots may have stimulated microbial decomposition of native SOC—a pattern consistent with a priming effect. Although we cannot directly quantify priming with our data these findings support the idea that root-derived inputs can accelerate the turnover of old carbon. Climatic conditions at our study site (mild winters, high precipitation) may have further contributed to sustained microbial activity and mineralization across the profile, in contrast to the grassland site in Colorado (USA) where the original soil was collected friom and where colder conditions could limit decomposition. However, the formation of new soil organic matter was more pronounced in the topsoil, leading to a net increase in carbon storage at the surface but not at 50 cm depth. This pattern suggests that while microbial activity and decomposition were not strongly depth dependent, organic inputs – primarily from plant residues and root exudates – were concentrated near the surface. One limitation of our study is the discontinuous depth intervals which prevented us from precisely identifying where along the profile these changes occurred. These depth jumps were a necessary trade-off due to cost constraints, but future studies with finer sampling resolution would provide more detailed insights into SOC distribution. Although we cannot determine the exact depth at which the changes occurred due to the discontinuous depth intervals, our results indicate that the shift is likely to happen between 30 and 50 cm.

**Fig. 9:**
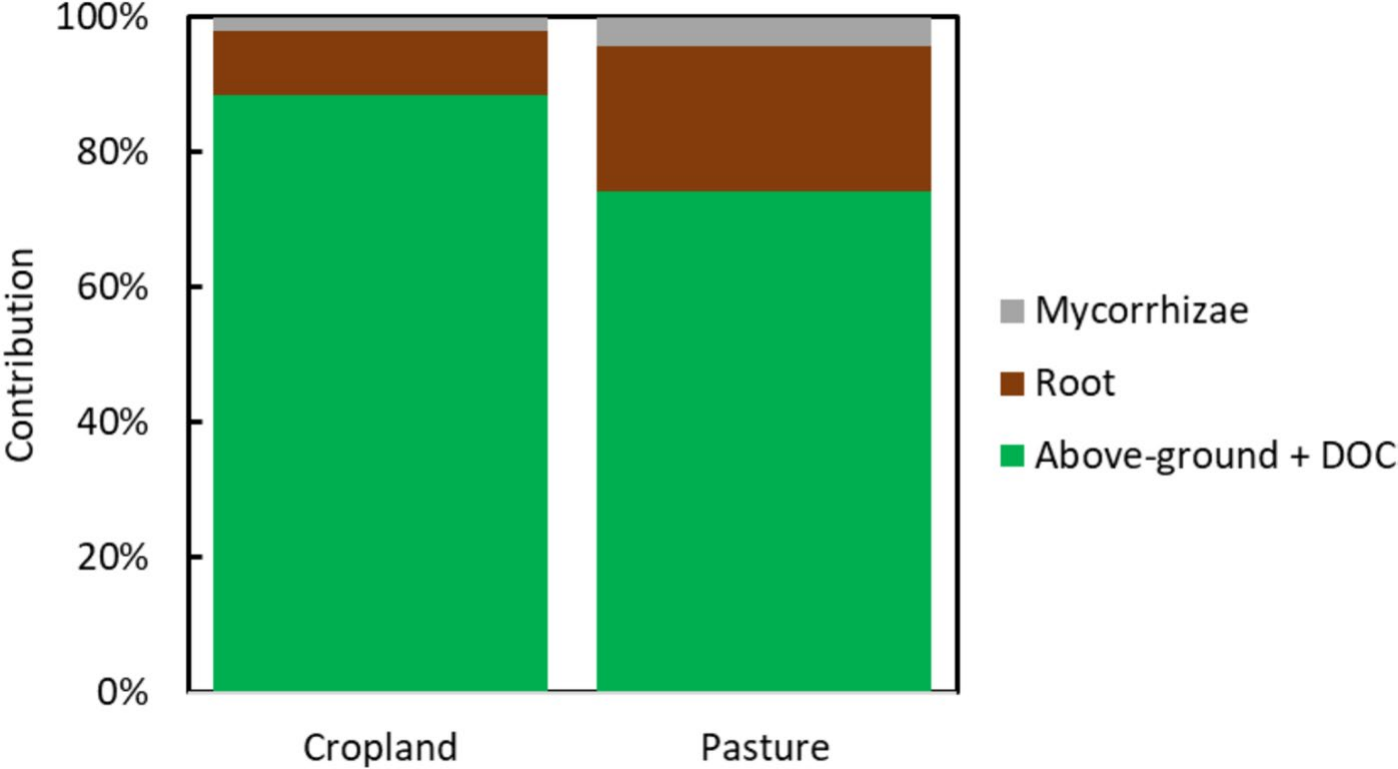
Relative contributions of C inputs from poplars: above-ground litter and dissolved organic carbon (DOC), roots, and mycorrhizae to the formation of new soil organic carbon (SOC) in the former cropland and pasture land uses. Data are shown as percentages

Mycorrhizae play an important role in the global carbon cycle, accounting for approximately 13% of net primary production (NPP) in ectomycorrhizal plants (Hawkins et al. 2023). Poplar species are known to form both ectomycorrhizal (ECM) and arbuscular mycorrhizal (AM) associations (Szuba 2015; Godbold et al. 2006), and ECM in particular have been identified as a dominant pathway for carbon input into soils under elevated CO₂ conditions (Godbold et al. 2006). Based on these earlier studies our second hypothesis was that mycorrhizal fungi are more important for carbon accumulation in the soil than fine roots. However, the treatment allowing root colonization consistently showed the highest amounts of poplar-derived carbon at all depths compared to the mycorrhizae treatment, highlighting the dominant role of roots in contributing to soil carbon accrual through processes such as root turnover and rhizodeposition. Thus, our second hypothesis was rejected. Moreover, above-ground and DOC contribution exceeded the contribution of roots and mycorrhizae (Fig. 9). Data from a study using biomarkers in successional subtropical forests in China showed that rapid SOC accumulation during the early- to mid-successional stages (25 to 55 years) was driven jointly by root- and microbe-derived carbon inputs (Liu et al. 2022). In contrast, above-ground litter fall significantly contributed to SOC accrual during the mid- to late-successional stages (55 to 120 years). Although subtropical forests are not directly comparable to a coppice system, the findings highlight the crucial role of root production and microbial anabolism in SOC accrual during the early stages of forest succession, while emphasizing the long-term contribution of above-ground leaf litter to topsoil SOC accrual (Liu et al. 2022). In tropical montane forests in Brazil, ecosystems that have experienced greater disturbances tend to allocate more resources to fine-root production while less carbon is invested in arbuscular mycorrhizal fungi (da Silva et al. 2020). These insights on ecosystems of considerably higher lifetime emphasize the critical role of root systems in carbon cycling, suggesting that enhancing root biomass through management practices could further improve soil carbon accrual in various ecosystems. In contrast to our previous study where roots were the main contributor in the short term (Berhongaray et al. 2019), above-ground and DOC carbon input played a more important role in the longer term. Moreover, these inputs appeared to influence SOC dynamics differently across depth – with root-derived SOC likely contributing more to deeper soil layers – while above-ground and DOC inputs primarily affected the surface, emphasizing the need to consider both spatial and temporal scales when assessing SOC accrual.

Our study also revealed that the in-growth cores were partially covered by a thin layer of C_3_ soil, potentially contributing additional carbon through DOC leaching. Assuming a SOC concentration of 2% and a BD of 1.4 kg m^−3^, this layer could have provided approximately 0.28 kg C m^−2^. Considering that total SOC gains in the control treatment reached 1.4 kg C m^−2^, DOC from this surface soil layer could account for approximately 20–40% of the new carbon detected in the in-growth cores. This suggests that some of the SOC previously attributed to above-ground litter and DOC inputs may in fact include contributions from DOC leached from this soil layer. Alternatively, this DOC flux can be seen as an integral component of SOC dynamics in systems with elevated initial SOC stocks, underscoring the need to carefully account for this process when quantifying new carbon inputs.

Recent research indicates that arbuscular mycorrhizal fungi can enhance SOC decomposition by unlocking mineral elements and promoting the formation of reactive minerals, thereby reducing mineral-associated carbon (Li et al. 2023a). The observed increase in SOC in the control treatment supports our third hypothesis that”the presence of roots accelerates the mineralisation of soil organic matter, but this carbon loss is outweighed by the formation of new soil organic matter”. So, this illustrates the complex interaction between root induced mineralization and new organic matter formation. The accelerated mineralization likely resulted from increased enzyme activity and microbial stimulation associated with root exudates and decomposition (Jiang et al. 2021; Li et al. 2023b). However, this increased root activity might not be solely catabolic. Simultaneously, substantial inputs of new carbon from root turnover and rhizodeposition more than compensated for the losses due to mineralization (Fig. 8). Therefore our findings confirm that, while roots stimulate mineralization, the resulting positive carbon balance underscores their overall contribution to SOC accrual. However, when analyzing the plantation soils directly) – that is, through field-based soil cores outside the in-growth bags – this impact varied significantly between former croplands and pastures. Only the former cropland showed increased SOC as in the in-growth cores. Former pasture showed a decrease suggesting that carbon inputs were insufficient to offset losses from mineralization. Although we did not analyse the of r- and k-strategists of microbes on SOC decomposition they have an impact on SOC changes and storage. It is plausible that r-strategist microbes dominated the pre-plantation agricultural environment, which was characterized by episodic disturbances such as ploughing, manure application, and seeding. These r-strategists may have persisted after plantation establishment, taking advantage of seasonal peaks in litter input and occasional soil disturbance from coppicing. However, given the lack of direct evidence, we cannot elaborate further on this topic. Successful SOC accrual strategies therefore require considering pre-existing soil conditions. While carbon inputs are critical determinants of soil C levels (Berhongaray And Alvarez 2019), the lower levels observed in pasture suggest insufficient input compared to the cropland, highlighting a need for increased input in these areas — or alternatively, avoiding plantation establishment on carbon-rich grasslands to preserve existing stocks. Alternative managements, as intercropping switchgrass with hybrid poplar, which has demonstrated increased SOC accrual in sandy soils (Collins et al. 2020), could be used for enhanced SOC accrual in rich C soils as pasture land.

Our results suggest to crop SRC poplar preferentially on former cropland with low initial SOC (i.e., a measurable C-saturation deficit; Stewart et al. 2009) and avoiding conversion of C-rich pastures where new inputs may not offset stimulated mineralization. Management should (i) minimize disturbance at establishment; (ii) maintain surface litter to sustain the substantial above-ground/DOC contribution over time; (iii) favor genotypes/rotation schemes that promote fine-root turnover and rhizodeposition; and (iv) consider diversification (e.g., SRC with herbaceous intercrops) where additional surface inputs are desired. For policy and accounting, SOC monitoring should extend to at least 30–60 cm and over decadal timescales, and incentives should target low-SOC croplands where SRC can credibly deliver both biomass and verifiable SOC accrual.

## Conclusion

This study demonstrates that poplar bioenergy plantations can significantly contribute to soil carbon accumulation in carbon-poor agricultural soils, particularly in the upper soil layers. The inclusion of roots and mycorrhizae was key to maximizing new carbon inputs, although DOC and above-ground litter inputs also played an important role. In soils with higher initial SOC stocks, such as former pastures, carbon inputs were insufficient to offset losses from mineralization, highlighting the importance of considering pre-existing soil conditions when implementing bioenergy systems.

While roots are crucial drivers of carbon accumulation, the role of mycorrhizae and surface inputs should not be overlooked when designing management strategies aimed at improving soil carbon balance. These results reinforce the notion that bioenergy plantations on marginal croplands represent a promising tool for carbon sequestration, with direct implications for climate change mitigation policies. Future research should focus on better understanding the interaction between surface and below-ground carbon inputs across different soil types and on evaluating management strategies that optimize the balance between mineralization and SOC accumulation over the long term.

## Supplementary Information

Below is the link to the electronic supplementary material.

Supplementary file1 (DOCX 1357 KB)

## Data Availability

The datasets generated during and/or analysed during the current study are available from the corresponding author on reasonable request.
