# Supplementary material for: Root and mycorrhizal contributions to soil organic carbon changes following 12 years of poplar coppice on former cropland and grassland
Source: Plant Soil. 2025 Oct 29;518(1):231–50. doi: 10.1007/s11104-025-07995-2 (PMC12830469; doi:10.1007/s11104-025-07995-2)

**Suplementary Material**

SM 1: SOC sampling and soil ingrow-core management timing

Figure SM1.1: Schematic representation of the 2 + 2 + 3 + 2 + 2 + 2 rotation cycle of the SRC plantation, indicating the timing of SOC sampling and soil ingrowth-core installation and harvest. At the end of the second year of growth and at the end of each rotation all poplars were machine harvested by coppicing to 5-10 cm above ground level. The harvest of leafless poplar was always done in winter time. The harvested wood (stems and branches) were chipped and used for the production of bio-energy. At the onset of the next year after coppice all poplar stools resprouted with many shoots. These shoots undergo a self-thinning process; there was no intervention in the self-thinning. The difference between a two-year rotation and a three-year rotation is primarily in the diameter and the number of the shoots to be harvested. A full description of the coppice management has been provided by Broeckx et al. (2012) and Verlinden et al. (2015). Both publications have been listed in the Reference list.


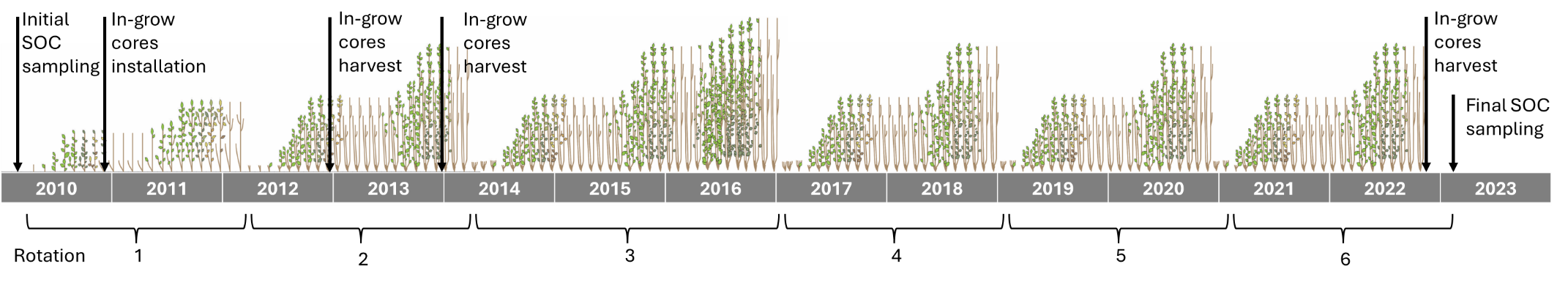


***SM 2: contribution of the different sources (leaves, wood and roots) to the total d13C litter input***

Average δ13C_Plant_ was calculated considering the relative contribution of the different sources (leaves, wood and roots) to the total litter input for each treatment and soil depth. n= number of samples, SD: standard deviation, Prop: proportion from the total C input.

| Genotype | LandUse | Treat. | Depth | Wood | | | | Leaves | | | | Roots | | | | Plant |  |
| --- | --- | --- | --- | --- | --- | --- | --- | --- | --- | --- | --- | --- | --- | --- | --- | --- | --- |
|  |  |  |  | n | d13C | SD | Prop | n | d13C | SD | Prop | n | d13C | SD | Prop | d13C | SD |
| Koster | Cropland | I | A | 20 | -28.2 | 0.6 | 0.2 | 20 | -28.8 | 0.72 | 0.7 | 18 | -28.9 | 0.4 | 0.1 | -28.7 | 0.7 |
| Koster | Cropland | I | B | 20 | -28.2 | 0.6 | 0 | 20 | -28.8 | 0.72 | 0 | 18 | -28.9 | 0.4 | 1 | -28.9 | 0.4 |
| Koster | Cropland | I | C | 20 | -28.2 | 0.6 | 0 | 20 | -28.8 | 0.72 | 0 | 18 | -28.9 | 0.4 | 1 | -28.9 | 0.4 |
| Koster | Cropland | II | A | 20 | -28.2 | 0.6 | 0 | 20 | -28.8 | 0.72 | 0 | 18 | -28.9 | 0.4 | 1 | -28.9 | 0.4 |
| Koster | Cropland | II | B | 20 | -28.2 | 0.6 | 0 | 20 | -28.8 | 0.72 | 0 | 18 | -28.9 | 0.4 | 1 | -28.9 | 0.4 |
| Koster | Cropland | II | C | 20 | -28.2 | 0.6 | 0 | 20 | -28.8 | 0.72 | 0 | 18 | -28.9 | 0.4 | 1 | -28.9 | 0.4 |
| Koster | Cropland | III | A | 20 | -28.2 | 0.6 | 0.25 | 20 | -28.8 | 0.72 | 0.75 | 18 | -28.9 | 0.4 | 0 | -28.6 | 0.7 |
| Koster | Cropland | III | B | 20 | -28.2 | 0.6 | 0.25 | 20 | -28.8 | 0.72 | 0.75 | 18 | -28.9 | 0.4 | 0 | -28.6 | 0.7 |
| Koster | Cropland | III | C | 20 | -28.2 | 0.6 | 0.25 | 20 | -28.8 | 0.72 | 0.75 | 18 | -28.9 | 0.4 | 0 | -28.6 | 0.7 |
| Koster | Cropland | IV | A | 20 | -28.2 | 0.6 | 0.25 | 20 | -28.8 | 0.72 | 0.75 | 18 | -28.9 | 0.4 | 0 | -28.6 | 0.7 |
| Koster | Cropland | IV | B | 20 | -28.2 | 0.6 | 0.25 | 20 | -28.8 | 0.72 | 0.75 | 18 | -28.9 | 0.4 | 0 | -28.6 | 0.7 |
| Koster | Cropland | IV | C | 20 | -28.2 | 0.6 | 0.25 | 20 | -28.8 | 0.72 | 0.75 | 18 | -28.9 | 0.4 | 0 | -28.6 | 0.7 |
| Koster | Pasture | I | A | 19 | -27.7 | 0.6 | 0.2 | 20 | -27.9 | 0.5 | 0.7 | 18 | -28.4 | 0.4 | 0.1 | -27.9 | 0.5 |
| Koster | Pasture | I | B | 19 | -27.7 | 0.6 | 0 | 20 | -27.9 | 0.5 | 0 | 18 | -28.4 | 0.4 | 1 | -28.4 | 0.4 |
| Koster | Pasture | I | C | 19 | -27.7 | 0.6 | 0 | 20 | -27.9 | 0.5 | 0 | 18 | -28.4 | 0.4 | 1 | -28.4 | 0.4 |
| Koster | Pasture | II | A | 19 | -27.7 | 0.6 | 0 | 20 | -27.9 | 0.5 | 0 | 18 | -28.4 | 0.4 | 1 | -28.4 | 0.4 |
| Koster | Pasture | II | B | 19 | -27.7 | 0.6 | 0 | 20 | -27.9 | 0.5 | 0 | 18 | -28.4 | 0.4 | 1 | -28.4 | 0.4 |
| Koster | Pasture | II | C | 19 | -27.7 | 0.6 | 0 | 20 | -27.9 | 0.5 | 0 | 18 | -28.4 | 0.4 | 1 | -28.4 | 0.4 |
| Koster | Pasture | III | A | 19 | -27.7 | 0.6 | 0.25 | 20 | -27.9 | 0.5 | 0.75 | 18 | -28.4 | 0.4 | 0 | -27.8 | 0.5 |
| Koster | Pasture | III | B | 19 | -27.7 | 0.6 | 0.25 | 20 | -27.9 | 0.5 | 0.75 | 18 | -28.4 | 0.4 | 0 | -27.8 | 0.5 |
| Koster | Pasture | III | C | 19 | -27.7 | 0.6 | 0.25 | 20 | -27.9 | 0.5 | 0.75 | 18 | -28.4 | 0.4 | 0 | -27.8 | 0.5 |
| Koster | Pasture | IV | A | 19 | -27.7 | 0.6 | 0.25 | 20 | -27.9 | 0.5 | 0.75 | 18 | -28.4 | 0.4 | 0 | -27.8 | 0.5 |
| Koster | Pasture | IV | B | 19 | -27.7 | 0.6 | 0.25 | 20 | -27.9 | 0.5 | 0.75 | 18 | -28.4 | 0.4 | 0 | -27.8 | 0.5 |
| Koster | Pasture | IV | C | 19 | -27.7 | 0.6 | 0.25 | 20 | -27.9 | 0.5 | 0.75 | 18 | -28.4 | 0.4 | 0 | -27.8 | 0.5 |
| Skado | Cropland | I | A | 20 | -26.9 | 0.5 | 0.2 | 20 | -27.0 | 0.4 | 0.7 | 18 | -28.0 | 0.7 | 0.1 | -27.1 | 0.4 |
| Skado | Cropland | I | B | 20 | -26.9 | 0.5 | 0 | 20 | -27.0 | 0.4 | 0 | 18 | -28.0 | 0.7 | 1 | -28.0 | 0.7 |
| Skado | Cropland | I | C | 20 | -26.9 | 0.5 | 0 | 20 | -27.0 | 0.4 | 0 | 18 | -28.0 | 0.7 | 1 | -28.0 | 0.7 |
| Skado | Cropland | II | A | 20 | -26.9 | 0.5 | 0 | 20 | -27.0 | 0.4 | 0 | 18 | -28.0 | 0.7 | 1 | -28.0 | 0.7 |
| Skado | Cropland | II | B | 20 | -26.9 | 0.5 | 0 | 20 | -27.0 | 0.4 | 0 | 18 | -28.0 | 0.7 | 1 | -28.0 | 0.7 |
| Skado | Cropland | II | C | 20 | -26.9 | 0.5 | 0 | 20 | -27.0 | 0.4 | 0 | 18 | -28.0 | 0.7 | 1 | -28.0 | 0.7 |
| Skado | Cropland | III | A | 20 | -26.9 | 0.5 | 0.25 | 20 | -27.0 | 0.4 | 0.75 | 18 | -28.0 | 0.7 | 0 | -27.0 | 0.4 |
| Skado | Cropland | III | B | 20 | -26.9 | 0.5 | 0.25 | 20 | -27.0 | 0.4 | 0.75 | 18 | -28.0 | 0.7 | 0 | -27.0 | 0.4 |
| Skado | Cropland | III | C | 20 | -26.9 | 0.5 | 0.25 | 20 | -27.0 | 0.4 | 0.75 | 18 | -28.0 | 0.7 | 0 | -27.0 | 0.4 |
| Skado | Cropland | IV | A | 20 | -26.9 | 0.5 | 0.25 | 20 | -27.0 | 0.4 | 0.75 | 18 | -28.0 | 0.7 | 0 | -27.0 | 0.4 |
| Skado | Cropland | IV | B | 20 | -26.9 | 0.5 | 0.25 | 20 | -27.0 | 0.4 | 0.75 | 18 | -28.0 | 0.7 | 0 | -27.0 | 0.4 |
| Skado | Cropland | IV | C | 20 | -26.9 | 0.5 | 0.25 | 20 | -27.0 | 0.4 | 0.75 | 18 | -28.0 | 0.7 | 0 | -27.0 | 0.4 |
| Skado | Pasture | I | A | 18 | -26.4 | 0.5 | 0.2 | 20 | -26.6 | 0.3 | 0.7 | 18 | -27.5 | 0.7 | 0.1 | -26.7 | 0.4 |
| Skado | Pasture | I | B | 18 | -26.4 | 0.5 | 0 | 20 | -26.6 | 0.3 | 0 | 18 | -27.5 | 0.7 | 1 | -27.5 | 0.7 |
| Skado | Pasture | I | C | 18 | -26.4 | 0.5 | 0 | 20 | -26.6 | 0.3 | 0 | 18 | -27.5 | 0.7 | 1 | -27.5 | 0.7 |
| Skado | Pasture | II | A | 18 | -26.4 | 0.5 | 0 | 20 | -26.6 | 0.3 | 0 | 18 | -27.5 | 0.7 | 1 | -27.5 | 0.7 |
| Skado | Pasture | II | B | 18 | -26.4 | 0.5 | 0 | 20 | -26.6 | 0.3 | 0 | 18 | -27.5 | 0.7 | 1 | -27.5 | 0.7 |
| Skado | Pasture | II | C | 18 | -26.4 | 0.5 | 0 | 20 | -26.6 | 0.3 | 0 | 18 | -27.5 | 0.7 | 1 | -27.5 | 0.7 |
| Skado | Pasture | III | A | 18 | -26.4 | 0.5 | 0.25 | 20 | -26.6 | 0.3 | 0.75 | 18 | -27.5 | 0.7 | 0 | -26.6 | 0.3 |
| Skado | Pasture | III | B | 18 | -26.4 | 0.5 | 0.25 | 20 | -26.6 | 0.3 | 0.75 | 18 | -27.5 | 0.7 | 0 | -26.6 | 0.3 |
| Skado | Pasture | III | C | 18 | -26.4 | 0.5 | 0.25 | 20 | -26.6 | 0.3 | 0.75 | 18 | -27.5 | 0.7 | 0 | -26.6 | 0.3 |
| Skado | Pasture | IV | A | 18 | -26.4 | 0.5 | 0.25 | 20 | -26.6 | 0.3 | 0.75 | 18 | -27.5 | 0.7 | 0 | -26.6 | 0.3 |
| Skado | Pasture | IV | B | 18 | -26.4 | 0.5 | 0.25 | 20 | -26.6 | 0.3 | 0.75 | 18 | -27.5 | 0.7 | 0 | -26.6 | 0.3 |
| Skado | Pasture | IV | C | 18 | -26.4 | 0.5 | 0.25 | 20 | -26.6 | 0.3 | 0.75 | 18 | -27.5 | 0.7 | 0 | -26.6 | 0.3 |

***SM 3: Map of the field site***

Map of the field lay-out showing the soil sample locations on both former land use types: cropland and pasture. The field area was reduced in 2023.


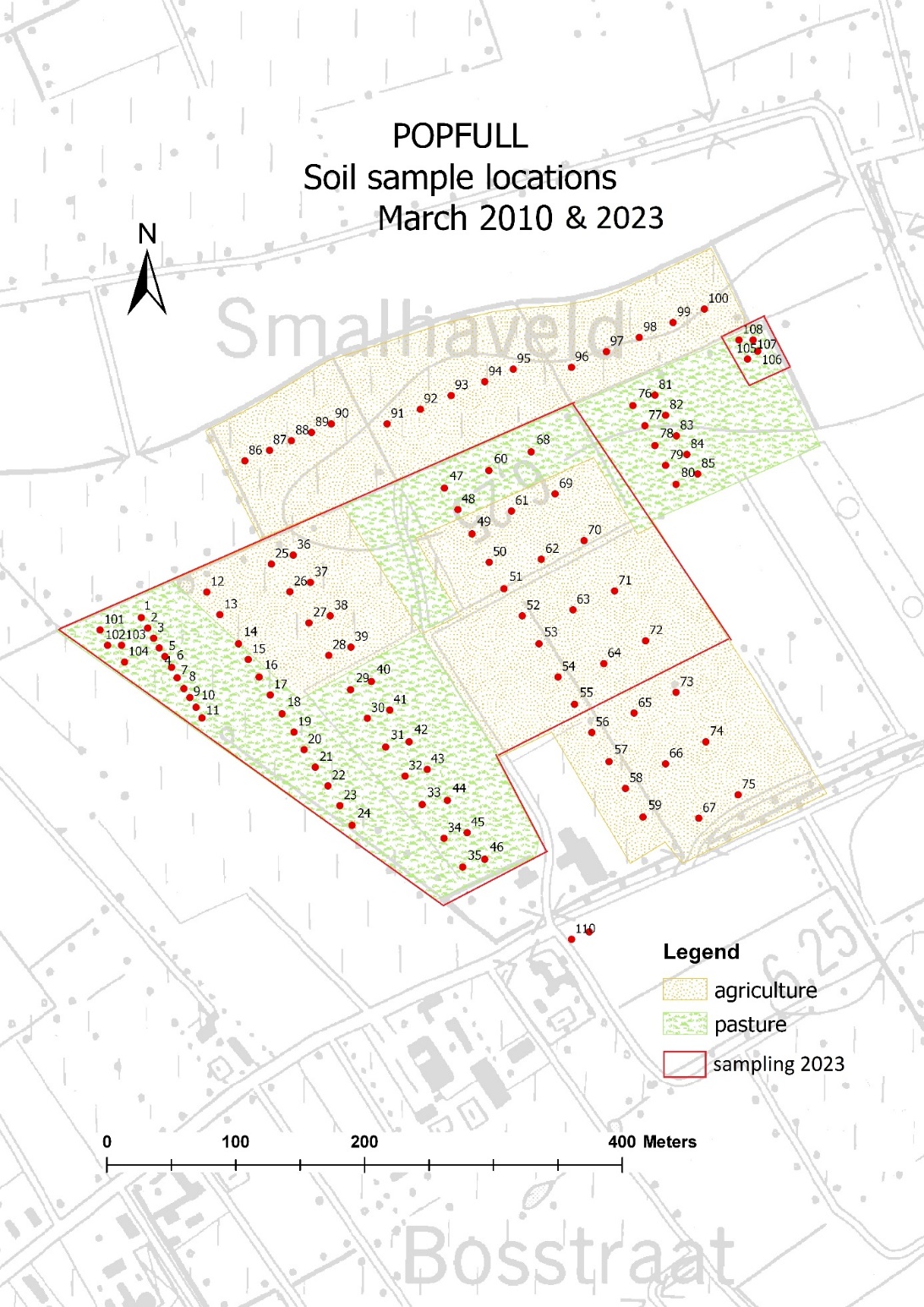


***SM 4:* Soil organic carbon (SOC) stocks (0–90 cm)**

No significant (p>0.05) changes in SOC stocks were observed between the two sampling years, indicating stable carbon content in the absence of poplar plantation. These data support the attribution of SOC changes in the main experiment to the effects of the plantation rather than background temporal variation.


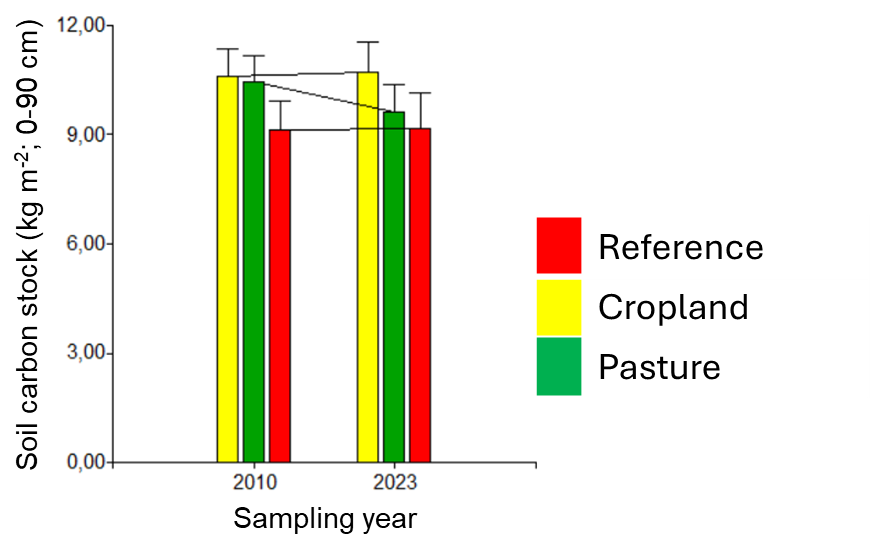


**Figure SM 4.** Soil organic carbon (SOC) stocks (0–90 cm) in the reference plots, croplands and pasture measured in 2010 and 2023. These “reference” unplanted control areas allow assessment of natural SOC variation over the 14-year period without tree plantation. Bars represent mean SOC stocks (kg C m⁻²) ± standard error for each of the three reference blocks (colored bars).

***SM 5:* Evolution of soil δ¹³C values from 2010 to 2022**

Soil δ¹³C values declined similarly across treatments in Skado–Cropland, whereas in Koster–Pasture, the exclusion of roots and/or mycorrhizae led to slower rates of δ¹³C change, suggesting a lower contribution of belowground inputs to SOC formation. These results highlight the long-term dynamics of C replacement and the role of fine roots and mycorrhizae in different genotype × land-use contexts.

**Figure SM 5.** The evolution of soil δ¹³C values from 2010 to 2022 is shown for the three treatments (control, Excluding roots, Excluding mycorrhiza) across both poplar genotypes (Koster and Skado) and former land uses (cropland and pasture). Across all panels, a consistent decrease in δ¹³C values was observed over time, reflecting the progressive incorporation of poplar-derived (C₃) carbon into the originally C₄-derived soil organic matter. Earlier time points (2010, 2012, and 2014) were reported previously in Berhongaray et al. (2019), while the final sampling from 2022 is presented here for the first time.

***SM 6 Statistics on SOC and SON at equivalent soil mass.***

**ANOVA – SOIL CARBON**

Soil mass (kg m⁻²) Variable N R² Adj R² CV

3300 Soil C stock (kg m⁻²) 65 1,00 0,52 13,82

**ANOVA table (type I SS)**

Factor SS df MS F p-value

Model 48,53 34 1,43 3,07 0,0012

SAMPLING YEAR 0,05 1 0,05 0,11 0,7397

FORMER LANDUSE 0,04 1 0,04 0,03 0,8680

FORMER LANDUSE>LOCATION ID.. 43,97 31 1,42 3,05 0,0015

SAMPLING YEAR*FORMER LANDU.. 4,46 1 4,46 9,59 0,0042

Error 13,95 30 0,46

Total 62,48 64

**Contrast**

SAMPLING YEAR*FORMER LANDU.. Contrast S.E. SS df MS F p-value

Contrast1 -0,62 0,00 2,41 1 2,41 5,18 0,0301

Contrast2 0,48 0,00 2,32 1 2,32 4,99 0,0332

Total 4,73 2 2,36 5,08 0,0125

**Contrast coefficients**

SAMPLING YEAR*FORMER LANDU.. Ct.1 Ct.2

2010:cropland 1,00 0,00

2010:pasture 0,00 1,00

2023:cropland -1,00 0,00

2023:pasture 0,00 -1,00

Soil mass (kg m⁻²) Variable N R² Adj R² CV

10700 Soil C stock (kg m⁻²) 65 1,00 0,72 23,22

**ANOVA table (type I SS)**

Factor SS df MS F p-value

Model 309,13 34 9,09 5,74 <0,0001

SAMPLING YEAR 2,69 1 2,69 1,70 0,2021

FORMER LANDUSE 5,56 1 5,56 0,57 0,4546

FORMER LANDUSE>LOCATION ID.. 300,65 31 9,70 6,13 <0,0001

SAMPLING YEAR*FORMER LANDU.. 0,22 1 0,22 0,14 0,7124

Error 47,50 30 1,58

Total 356,62 64

**Contrast**

SAMPLING YEAR*FORMER LANDU.. Contrast S.E. SS df MS F p-value

Contrast1 0,52 0,00 1,67 1 1,67 1,06 0,3121

Contrast2 0,36 0,00 1,27 1 1,27 0,80 0,3774

Total 2,94 2 1,47 0,93 0,4057

**Contrast coefficients**

SAMPLING YEAR*FORMER LANDU.. Ct.1 Ct.2

2010:cropland 1,00 0,00

2010:pasture 0,00 1,00

2023:cropland -1,00 0,00

2023:pasture 0,00 -1,00

**ANOVA – SOIL NITROGEN**

Soil mass (kg m⁻²) Variable N R² Adj R² CV

3300 Soil N stock (kg m⁻²) 65 1,00 0,55 15,03

**ANOVA table (type I SS)**

Factor SS df MS F p-value

Model 0,40 34 0,01 3,34 0,0006

SAMPLING YEAR 0,12 1 0,12 34,68 <0,0001

FORMER LANDUSE 4,2E-03 1 4,2E-03 0,63 0,4350

FORMER LANDUSE>LOCATION ID.. 0,21 31 0,01 1,90 0,0406

SAMPLING YEAR*FORMER LANDU.. 0,07 1 0,07 18,79 0,0002

Error 0,10 30 3,5E-03

Total 0,50 64

**Contrast**

SAMPLING YEAR*FORMER LANDU.. Contrast S.E. SS df MS F p-value

Contrast1 0,01 0,00 2,7E-04 1 2,7E-04 0,08 0,7840

Contrast2 0,14 0,00 0,18 1 0,18 52,67 <0,0001

Total 0,18 2 0,09 26,37 <0,0001

**Contrast coefficients**

SAMPLING YEAR*FORMER LANDU.. Ct.1 Ct.2

2010:cropland 1,00 0,00

2010:pasture 0,00 1,00

2023:cropland -1,00 0,00

2023:pasture 0,00 -1,00

Soil mass (kg m⁻²) Variable N R² Adj R² CV

10700 Soil N stock (kg m⁻²) 65 1,00 0,67 18,81

**ANOVA table (type I SS)**

Factor SS df MS F p-value

Model 0,80 34 0,02 4,90 <0,0001

SAMPLING YEAR 0,22 1 0,22 45,93 <0,0001

FORMER LANDUSE 0,03 1 0,03 1,80 0,1897

FORMER LANDUSE>LOCATION ID.. 0,55 31 0,02 3,68 0,0003

SAMPLING YEAR*FORMER LANDU.. 2,5E-04 1 2,5E-04 0,05 0,8206

Error 0,14 30 4,8E-03

Total 0,94 64

**Contrast**

SAMPLING YEAR*FORMER LANDU.. Contrast S.E. SS df MS F p-value

Contraste1 0,11 0,00 0,08 1 0,08 16,99 0,0003

Contraste2 0,12 0,00 0,14 1 0,14 29,62 <0,0001

Total 0,22 2 0,11 23,30 <0,0001

**Contrast coefficients**

SAMPLING YEAR*FORMER LANDU.. Ct.1 Ct.2

2010:cropland 1,00 0,00

2010:pasture 0,00 1,00

2023:cropland -1,00 0,00

2023:pasture 0,00 -1,00

***SM 7 Details of weed control***

This table summarizes the weed-management schedule applied at the SRC poplar site during the establishment year (2010) and the second growing season (2011), detailing the timing and type of each intervention (e.g., pre- and post-emergence herbicide applications and mechanical control) used to secure uniform stand establishment. The information was extracted from Broeckx (2013), Development of leaf area and above-ground biomass of different Populus genotypes in a bioenergy plantation (PhD thesis).

**Table SM 7:** Description and timing of weed control during the establishment year (2010) and the second growing season (2011). Extracted from Broeckx 2014.


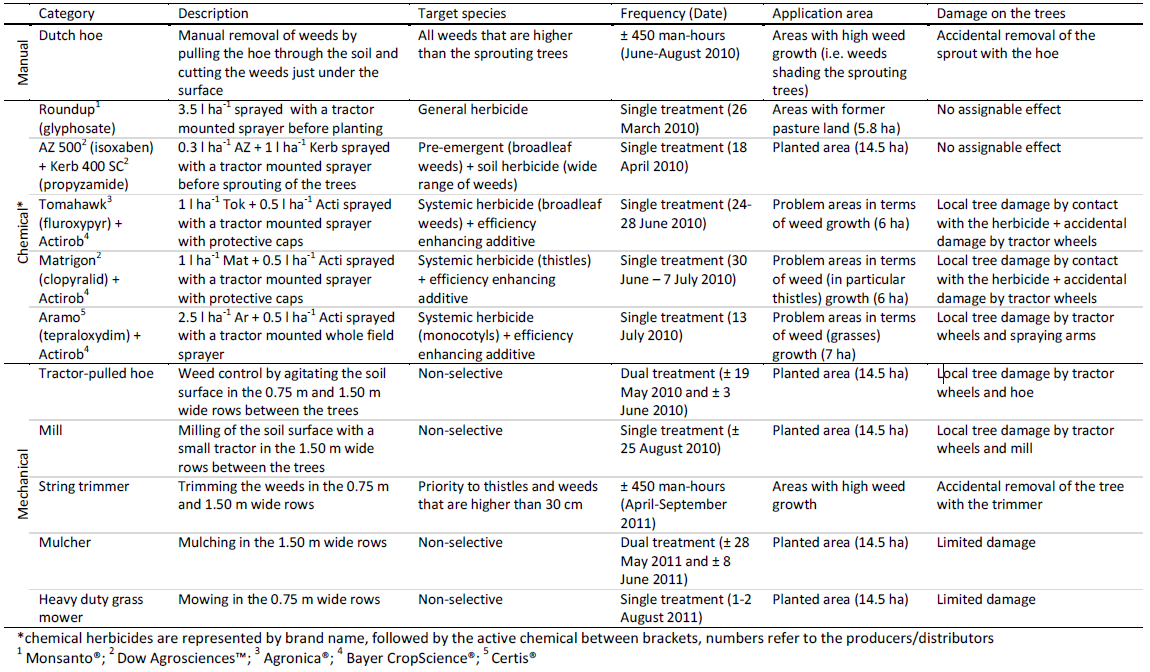

Supplement: Supplementary file 1 — Supplementary file1 (DOCX 1357 KB) [file 11104_2025_7995_MOESM1_ESM.docx]
